# Latent Anti-nutrients and Unintentional Breeding Consequences in Australian *Sorghum bicolor* Varieties

**DOI:** 10.3389/fpls.2021.625260

**Published:** 2021-03-01

**Authors:** Hayden E. Hodges, Heather J. Walker, Aaron J. Cowieson, Robert J. Falconer, Duncan D. Cameron

**Affiliations:** ^1^Department of Chemical and Biological Engineering, University of Sheffield, Sheffield, United Kingdom; ^2^biOMICS Facility, Faculty of Science, University of Sheffield, Sheffield, United Kingdom; ^3^DSM Nutritional Products, Kaiseraugst, Switzerland; ^4^Department of Chemical Engineering and Advanced Materials, University of Adelaide, Adelaide, SA, Australia; ^5^Department of Animal and Plant Science, University of Sheffield, Sheffield, United Kingdom

**Keywords:** *Sorghum bicolor*, metabolomics, Fourier-transform infrared, mass spectrometry, anti-nutrients, polyphenols, animal feed

## Abstract

Modern feed quality sorghum grain has been bred to reduce anti-nutrients, most conspicuously condensed tannins, but its inclusion in the diets of monogastric animals can still result in variable performance that is only partially understood. Sorghum grain contains several negative intrinsic factors, including non-tannin phenolics and polyphenols, phytate, and kafirin protein, which may be responsible for these muted feed performances. To better understand the non-tannin phenolic and polyphenolic metabolites that may have negative effects on nutritional parameters, the chemical composition of sorghum grain polyphenol extracts from three commercial varieties (MR-Buster, Cracka, and Liberty) was determined through the use of an under-studied, alternative analytical approach involving Fourier-transform infrared (FT-IR) spectroscopy and direct ionization mass spectrometry. Supervised analyses and interrogation of the data contributing to variation resulted in the identification of a variety of metabolites, including established polyphenols, lignin-like anti-nutrients, and complex sugars, as well as high levels of fatty acids which could contribute to nutritional variation and underperformance in monogastrics. FT-IR and mass spectrometry could both discriminate among the different sorghum varieties indicating that FT-IR, rather than more sophisticated chromatographic and mass spectrometric methods, could be incorporated into quality control applications.

## Introduction

Intensification of the global meat industry has stimulated innovation in the animal feed sector. Advances in feed technology strive towards intensification through a reduction in feed conversion ratio (FCR), greater energy and nutrient utilization, improved animal welfare and environmental sustainability, reduction in endogenous grain anti-nutrients, and optimization of costs (Makkar and Ankers, 2014). Using poultry production as a specific example, the yield of chicken meat (with Australia as a model market) has exponentially increased from the 1970s until today where it has begun to level off (Supplementary Figure S1). This increase in production is mirrored by a similar decrease and leveling off of FCR (increased efficiency). This gain of efficiency is due to several global innovations, including directed poultry and feed grain breeding, implementation of new feed additives, and use of higher quality grains and supplements in dietary formulations (Mottet and Tempio, 2017). Supplementation of monogastric feed with exogenous enzymes has become routine to support measures of performance, as well as to mitigate the effects of anti-nutrients, most commonly phytate, non-digestible starches and proteins, and polyphenolic compounds (Cowieson et al., 2006). Polyphenols are well-established anti-nutrients and antifeedants, particularly to monogastrics, and routinely cause reduced feed intake and weight gain, increased FCR (reduced efficiency), and enzyme inhibition (Bravo, 1998; Cadogan and Finn, 2010; Pasquali et al., 2016; Alu’datt et al., 2018).

While phenolic and polyphenolic compounds are found in all feed grains, sorghum, *Sorghum bicolor* (L.), is well-established as having markedly high levels of these secondary metabolites, including condensed tannins (Glennie et al., 1981; Awika and Rooney, 2004). Sorghum is the fifth-most important cereal crop grown in the world and has many diverse applications, including alcoholic beverages, biofuel, human food products, and animal feed. Approximately, 59 million tons of sorghum was produced in 2018 with half of the production in Africa and a third in the Americas (Food and Agricultural Organization of the United Nations, 2020). As sorghum has high levels of phenolic and polyphenolic compounds, it has developed a split nature as these metabolites have proven positive effects in human diets. The polyphenols found in sorghum are well-established antioxidants that can reduce oxidative stress and the diseases that arise from imbalances in reactive oxygen species (Awika and Rooney, 2004; Stefoska-Needham et al., 2015).

The higher concentrations of polyphenols, up to 10% of the grain’s mass, have played a key role in sorghum being stigmatized as having lower nutritional quality when incorporated into monogastric animal feed (Jambunathan and Mertz, 1973; Armstrong et al., 1974; Bravo, 1998; Selle et al., 2017). Previous studies on sorghum have identified a diverse range of phenolics, from small ferulic and caffeic acids to large condensed tannins with high degrees of polymerization (Stafford, 1965; Gupta and Haslam, 1978; Kang et al., 2016). High-tannin sorghum varieties are not commonly used in monogastric animal feed as deleterious nutritional effects have been observed in animals fed these particular grains (Nyachoti et al., 1996). These negative effects have encouraged sorghum breeders to preferentially select low-tannin varieties for use in monogastric animal feed. Sorghum grains low in tannin content, such as most red and white varieties in use today, have also been reported as having higher levels of digestible protein (Youssef, 1998). White sorghum grain, most commonly the Liberty variety in Australia, has been found to better support weight gain, FCR, and growth performance in pigs and chickens than its red colored counterparts. This may be due to the absence of large polyphenols, such as condensed tannins (Cadogan and Finn, 2010; Liu et al., 2010).

Currently, there is discussion about whether modern varieties, important to the animal feed industry, contain relevant/detectable levels of condensed tannins (Perez-Maldonado and Rodrigues, 2009; Liu et al., 2015). This debate seeks to move the conversation from condensed tannins to smaller phenolic and polyphenolic compounds which may contribute subtle differences in varietal performance, even in sorghum grains designated “tannin-free.” In their study of six “tannin-free” sorghum diets, Truong et al. (2016) found no difference in broiler chicken performance with regard to FCR and weight gain but did find differences in nutrient utilization between white and red grains. Negative correlations were found between phenolic acids, flavonols, kafirin protein (sorghum’s major storage protein), and measures of digestibility. Even the beneficial impacts of certain feed additive enzymes have been reported to be muted when formulated into sorghum diets. This has been observed primarily with phytase, with regard to the enzyme’s extra-phosphoric effects on protein and amino acid digestibility, (Liu et al., 2014; Truong et al., 2014; Selle et al., 2017). As the grains used in these studies were “tannin-free,” compounds other than traditional condensed tannins may have caused the anti-nutritional effects observed.

The majority of sorghum phenolic analyses have used liquid chromatography-mass spectrometry (LC-MS) with identifications achieved through use of standards, retention time comparison, and MS^n^ fragmentation (Kang et al., 2016; Rao et al., 2018; Tugizimana et al., 2019; Jiang et al., 2020; Zhou et al., 2020). The use and comparison of less intensive methodological approaches, including direct ionization and infrared spectroscopy, has been little studied in sorghum, especially with regard to characterizing metabolic variation between grain varieties important to the animal feed industry. Currently, there exists no comparative framework for the assessment of orthogonal methods of analysis for polyphenolic extracts, particularly crude extracts, from feed-relevant sorghum grains. In this paper, we present an alternative analytical framework for characterizing phenolic anti-nutrients in crude polyphenol extracts from three Australian sorghum varieties (MR-Buster, Cracka, and Liberty). Using a series of analytical techniques from simple spectroscopy to more complicated mass spectrometric methods, untargeted and targeted metabolomics methodologies were applied to the data to determine both bulk and subtle differences in metabolite profiles.

## Materials and Methods

### Materials

The sorghum varieties, MR-Buster, Cracka, and Liberty, were provided by DSM Nutritional Products (Kaiseraugst, Switzerland) and harvested in February 2017 from Central Darling Downs, Queensland, Australia. Solvents used were of high-performance liquid chromatography (HPLC) grade.

### Preparation of Sorghum Polyphenol Extracts

Sorghum grain was extracted for polyphenols following Harbertson et al. (2014) with modifications. Approximately 20 g of each variety were soaked overnight in ultra-high purity (UHP) water. The soaked grain was ground in a mortar and pestle and rinsed with UHP water six times, and allowed to dry overnight at room temperature. The dried bran was defatted for 4 h with 200 ml of *n*-hexane in a Soxhlet extractor. The defatted bran was allowed to dry overnight at room temperature prior to being extracted twice with 200 ml 70% (v/v) aq. acetone for 30 min on an orbital mixer (170 rpm). The acetone extract was filtered through glass filter paper, solvent removed in a rotary evaporator, lyophilized, and stored under nitrogen gas at −80°C. Three separate extracts were prepared per sorghum variety.

### Fourier Transform – Infrared Spectroscopy

Fourier-transform infrared spectroscopy (FT-IR) was performed on an IRAffinity-1S spectrometer (Shimadzu) using a diamond attenuated total reflectance (ATR) crystal (Specac Quest) in the wavenumber region between 4,000 and 400 cm^−1^ with a resolution of 4 cm^−1^ using Happ-Genzel Apodization. At each position, 40 scans were averaged. The spectra were baseline corrected with IR Solutions software (Shimadzu). Three separate extracts from each variety were each analyzed in triplicate and replicates were averaged. The spectra obtained from the sorghum polyphenol extracts were then analyzed for polyphenol and tannin structural features based on published spectra (Laghi et al., 2010; Falcão and Araújo, 2013, 2014; Ricci et al., 2016).

### Mass Spectrometry

Electrospray ionization [ESI; negative (−) and positive modes (+)] and matrix-assisted laser desorption/ionization (MALDI; +) mass spectrometry (MS) were performed on a Waters Synapt G2-Si ToF mass spectrometer (Waters Corporation, United States). MassLynx data system (Waters Corporation, United States) provided instrument control, data acquisition, and data processing. For all three analyses, sorghum polyphenol extracts were prepared to a concentration of 0.1 mg/ml in 50% (v/v) aq. methanol. Sorghum polyphenol extracts were prepared, run, and analyzed in triplicate and three different extracts per variety were analyzed. For ESI, capillary voltage was 2.2 kV, source temperature was 100°C, and desolvation temperature was 280°C. Solutions were injected at a flow rate of 5 μl min^−1^. ESI – tandem MS (MS^2^) was performed on specific ions produced by ESI-MS in the negative mode. For MALDI sample preparation, the matrix chemical alpha-cyano-4-hydroxycinnamic acid [CHCA; 5 mg/ml in methanol with 0.1% formic acid (v/v)] was mixed with the prepared extracts in a 1:1 ratio. From this mixture, 1 μl was spotted onto a steel MALDI plate for analysis. All spectra were measured from 50 to 1,500 Da for each analysis type.

### Data Processing and Statistical Analysis

Raw spectra data from each mass spectrometric analysis were processed following a stepwise method based on Overy et al. (2005) and Austen et al. (2019). Briefly, the raw mass spectrometry data were centroided and converted into text files using an in-house Visual Basic macro. The triplicate runs of each sample were combined to determine the average mass-to-charge ratio (*m/z*) of each compound ion to make-up the metabolite profile for each sample. The masses determined, along with their respective percent total ion count (TIC), were based on equations defined by Overy et al. (2005). For ease of analysis, masses were grouped together into “mass bins” based on groupings of 0.2 amu.

Principal component analysis (PCA) and orthogonal partial least squares discriminant analysis (OPLS-DA) were performed on the spectra obtained from FT-IR and the mass bins identified from the MS spectra using SIMCA (Sartorius Stedim Biotech, Sweden). PCA allows for the unsupervised, or untargeted, analysis of the metabolite profiles in the extracts which enables the separation of extracts based on metabolite variations among them. PCA provided the initial overview of the data to determine relationships between extract types and to highlight whether further investigation with more targeted analyses was needed. A covariance matrix was utilized over a correlation matrix as the data sets for each PCA were single-source and of the same data type (relative abundance units for FT-IR and percent TIC for mass spectrometry) and normalized using Pareto scaling prior to analysis. OPLS-DA is a supervised, or targeted, analysis which allows for pairwise comparisons to be made between two different extract types. This analysis maximizes variation between samples and produces quantitative loadings plots which highlight components of the spectra responsible for causing variation, i.e., wavenumbers (cm^−1^) from the FT-IR spectra and mass bins from the MS spectra. OPLS-DA was performed between MR-Buster and Cracka, MR-Buster and Liberty, and Cracka and Liberty.

For MS spectra, the top 10 mass bins causing variation for each extract in each pairing, as well as the 10 most abundant peaks, were interrogated further for putative identifications. Compound identification was conducted using online databases, including METLIN (Scripps Research Institute; La Jolla, CA, United States; https://metlin.scripps.edu) and Kyoto Encyclopedia of Genes and Genomes (KEGG; Kanehisa Laboratories; Kyoto, Japan; https://www.kegg.jp; Kaneshisa and Goto, 2000). In the negative mode, compounds were identified having an ion adduct of -H (−1.008 Da) while in positive mode ion adducts included +H (+1.008 Da), +Na (+22.99 Da), and +K (+39.10 Da). Following identifications, the KEGG IDs for all possible identifications in each mass bin were analyzed using MetaboAnalyst^1^ through the pathway analysis function with *Arabidopsis thaliana* as the pathway library, hypergeometric test as the over representation analysis, and relative-betweeness centrality for the pathway topology analysis (Chong et al., 2019).

The guidelines for compound identification were made following the guidance of the Chemical Analysis Working Group and the Metabolomics Standards Initiative (Sumner et al., 2007). These guidelines allow for four levels of identification of metabolites: (1) identified compound with two independent orthogonal data compared with an authentic sample; (2) putatively annotated compound relying on literature or database comparison; (3) putatively characterized compound classes; and (4) unknown compounds. The data obtained from FT-IR analysis are classified as a level 3 identification as established structural features of compound classes can be clearly identified. The identifications through mass spectrometry are classified as a level 2 identification and were accepted if below an *m/z* margin of error of 40 ppm or less.

Chicken yield data in Supplementary Figure S1 were plotted as individual values and an asymmetric sigmoidal 5 PL nonlinear model was fitted to the data using GraphPad Prism 8 (GraphPad Software, Inc., San Diego, CA, United States). The same was done to the FCR values collected from the literature, except the same nonlinear model was fitted to average values for each year rather than individual values.

## Results

### Qualitative Analysis of Sorghum Polyphenol Extract FT-IR Spectra

**Figure 1:**
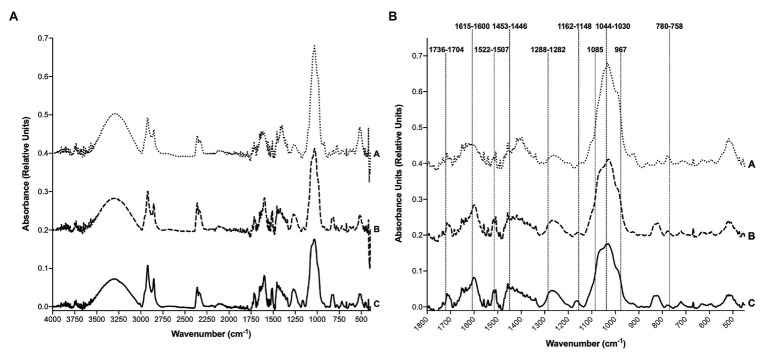
Fourier-transform infrared (FT-IR) spectra of sorghum polyphenol extracts. FT-IR spectra were obtained from **(A)** 4,000–400 cm^−1^ and **(B)** the fingerprint region from 1,800 to 450 cm^−1^. Three replicate spectra were averaged for each extract type. A, Liberty; B, Cracka; and C, MR-Buster. For more detail, see Supplementary Figures S2, S3.

Fourier-transform infrared spectroscopy was performed on the sorghum polyphenol extracts. The full spectra of the sorghum extracts matched closely to one another (Figure 1A). Cracka and MR-Buster spectra were essentially identical, while the Liberty extract spectrum displayed slight variations in peak location, size, and intensity. All extracts showed the presence of a hydroxyl (O – H) functional group marked by the presence of a strong, broad peak centered around 3,300–3,200 cm^−1^. The sorghum extracts displayed a weak, single peak/shoulder at approximately 3,010 cm^−1^ indicative of an aromatic C – H functional group. The sharp, strong peaks present in the spectra from 2,957 to 2,848 cm^−1^ are representative of an aliphatic C – H functional group. Within the fingerprint region (1,800–450 cm^−1^), 10 bands/peaks common to published tannin and polyphenol FT-IR spectra were highlighted in the spectra of the sorghum polyphenol extracts (Figure 1B). All sorghum extracts matched three of these highlighted wavenumber regions (1,736–1,704 cm^−1^, 1,044–1,030 cm^−1^, and 780–758 cm^−1^). The two red sorghum extracts, MR-Buster and Cracka, matched closely with another three regions (1,615–1,600 cm^−1^, 1,522–1,507 cm^−1^, and 1,162–1,148 cm^−1^). The four regions of the spectra not closely matched with any sorghum extract were 1,453–1,446 cm^−1^, 1,288–1,282 cm^−1^, 1,085 cm^−1^, and 967 cm^−1^.

### Multivariate Analysis (PCA, OPLS-DA) of Sorghum Polyphenol Extract FT-IR Spectra

**Figure 2:**
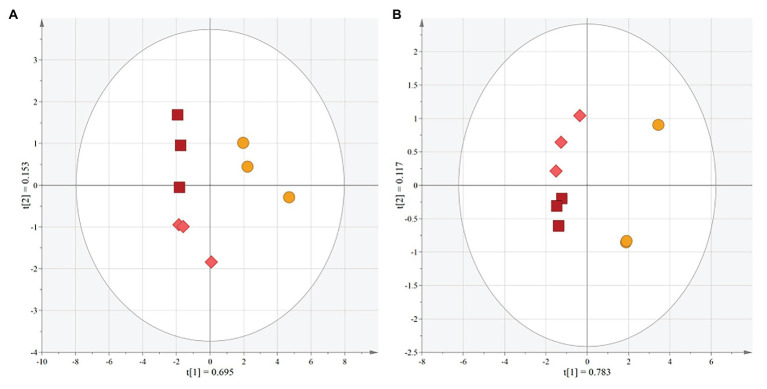
Principal component analysis (PCA) scores plots for FT-IR spectra from sorghum polyphenol extracts. PCA was performed on the full spectra (**A**: 4,000–400 cm^−1^) and fingerprint region (**B**: 1,800–450 cm^−1^) to determine relationships and variance between red and white sorghum polyphenol extracts. The ellipse represents a 95% CI. t(1) and t(2) represent principal components 1 and 2, respectively. MR-Buster (□) is dark red, Cracka (◇) is light red, and Liberty (◯) is yellow.

Multivariate analytical methods were applied to the FT-IR spectra using unsupervised PCA to determine if there was variation among extract types (Figures 2A,B). MR-Buster and Cracka extracts were clearly differentiated from Liberty extract in each of the analyses. The first two principal components of the sorghum polyphenol extracts explained 84.8% of the variation for the full spectra and 90% for the fingerprint region (1,800–450 cm^−1^). Supervised multivariate analysis was then conducted, using OPLS-DA, on the fingerprint regions of the FT-IR spectra to determine specific wavenumbers (cm^−1^) responsible for variation between extract types (Table 1; Supplementary Figure S4). OPLS-DA highlighted regions of the spectra most responsible for variations between pairwise comparisons of the sorghum extracts. Red sorghum (MR-Buster, Cracka) extracts were most varied in the regions corresponding to aromatic C – H (800s cm^−1^) and aromatic C = C bonds (1,600 cm^−1^), while white grain (Liberty) extract was most different in the aromatic C – H region (1,000–900 cm^−1^) and C – O bonding (1,030s cm^−1^).

**Table 1:** FT-IR wavenumbers (cm^−1^) identified from OPLS-DA loadings plots.

	MR-Buster (Red)	Cracka (Red)	Liberty (White)
MR-Buster (Red)	N/A	1,167–1,173 1,462 1,641–1,643 1,657–1,161	831–837 1,593–1,603
Cracka (Red)	1,055–1,074	N/A	829–837 1,595–1,601
Liberty (White)	988–997 1,030–1,034 1,045	984–997 1,034 1,045	N/A

### Unsupervised Analysis (PCA) of Sorghum Polyphenol Extract Metabolite Profiles From Mass Spectrometry

**Figure 3:**
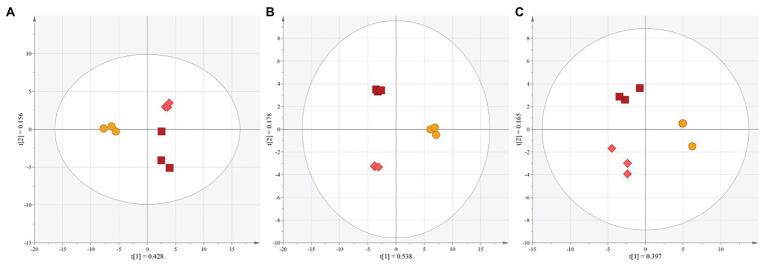
PCA scores plots of mass spectrometric analyses of polyphenol extracts. Unsupervised analyses were performed on data collected using **(A)** ESI (−), **(B)** ESI (+), and **(C)** MALDI (+) to determine relationships between red and white sorghum polyphenol extracts. The ellipse represents a 95% CI. t(1) and t(2) represent principal components 1 and 2, respectively. MR-Buster (□) is dark red, Cracka (◇) is light red, and Liberty (◯) is yellow.

Mass spectrometry was performed using ESI (+, −) and MALDI (+) (Supplementary Figure S5). Unsupervised analyses, using PCA, of the spectra allowed for the clear separation of red sorghum (MR-Buster, Cracka) and white sorghum (Liberty) extracts (Figures 3A–C). The first two principal components for ESI (−), ESI (+), and MALDI (+) explained 58.4, 71.6, and 56.2% of variation among sorghum extracts. These results indicated enough separation and variation between extract types to justify further supervised analyses to quantitatively determine specific mass bins, and thus metabolites, responsible for the variation in the extracts.

### Supervised Analysis (OPLS-DA) of Sorghum Polyphenol Extract Metabolite Profiles From Mass Spectrometry

**Figure 4:**
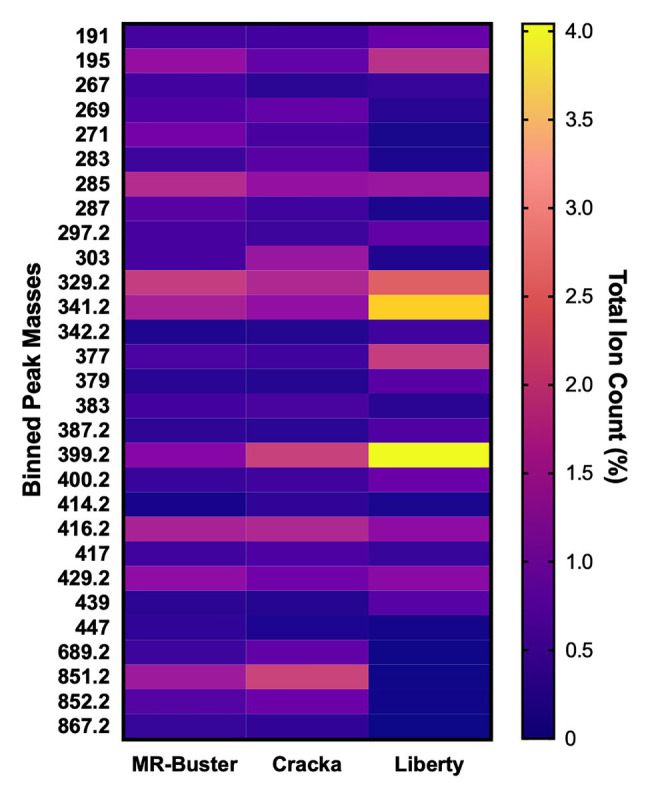
Heat map of percent total ion counts (TICs) for compounds identified from orthogonal partial least squares discriminant analysis (OPLS-DA). OPLS-DA (ESI [−]) indicated mass bins (*m/z*) most responsible for variation between pairwise comparisons of extracts. The mean relative abundance (total % ion count) and SD were formatted into a heat map, *n* = 3 for each mass bin (*m/z*).

Supervised analysis of the sorghum polyphenol extract spectra was performed using OPLS-DA, and pairwise comparisons were made between extract types. Data were binned to 0.2 amu chunks to minimize the amount of data handling and corresponding loadings plots were used to select mass bins (top 10) causing variation between extract pairings (Figure 4; Table 2; Supplementary Tables S1, S2; Supplementary Figures S6–S8). The identified mass bins from ESI (−) were then interrogated and identified using metabolite databases, including METLIN and KEGG (Supplementary Table S3). ESI-MS^2^ (−) was performed on select ions and found to match METLIN spectra and/or previous MS^2^ studies on sorghum polyphenol extracts (Kang et al., 2016; Supplementary Table S4).

**Table 2:** Binned peak masses identified from ESI (−) OPLS-DA loadings plots.

	MR-Buster (Red)	Cracka (Red)	Liberty (White)
MR-Buster (Red)	N/A	271 429.2 285 195 383 287 267 329.2 447 468.2	851.2 271 852.2 287 269 689.2 303 285 867.2 417
Cracka (Red)	399.2 416.2 303 851.2 383.2 689.2 283 852.2 414.2 269	N/A	851.2 303 852.2 689.2 269 283 417 271 383.2 416.2
Liberty (White)	399.2 341.2 377 400.2 379 329.2 387.2 439 191 342.2	341.2 399.2 377 329.2 379 439 400.2 191 297.2 387.2	N/A

**Figure 5:**
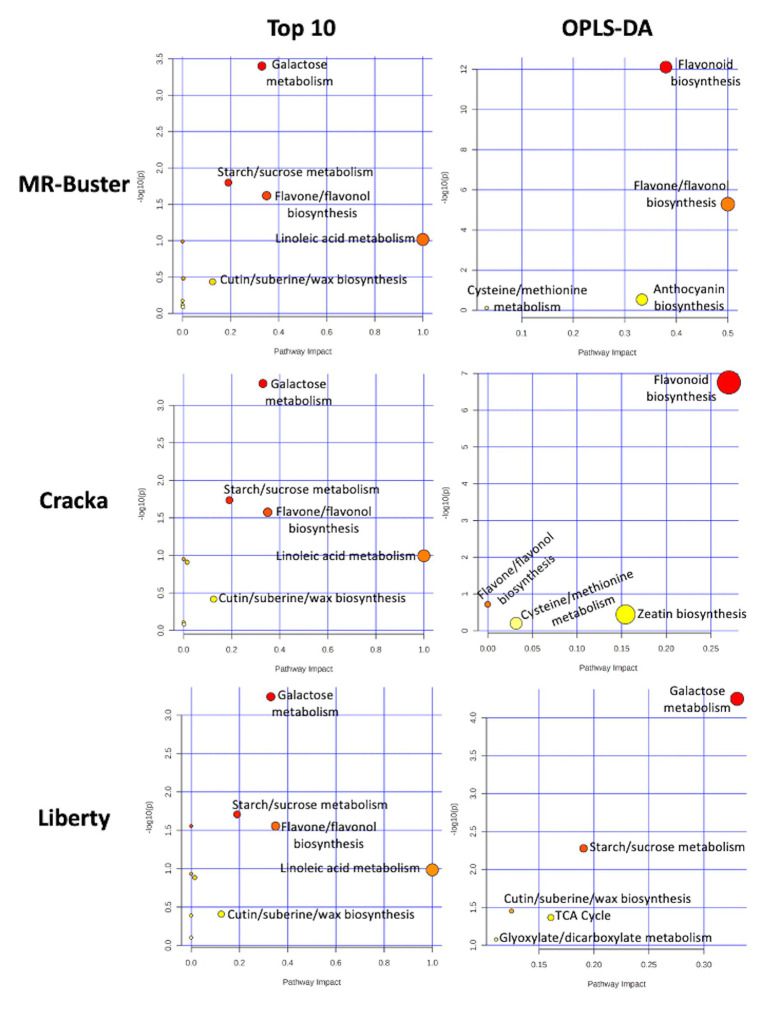
Pathway analysis of identified compounds from ESI (−). Mass bins (*m/z*) representing the 10 most abundant ions and those highlighted as causing variance between sorghum extracts were identified and mapped to biosynthetic pathways using MetaboAnalyst Pathway Analyst. This analysis identified the most relevant biosynthetic pathways associated with the compounds identified. The pathways were then ranked based on their impact with a value closer to one (red) as being more impactful than a value closer to zero (yellow). The *y*-axis, −log(10)p, is a measure of statistical significance.

The mass bins responsible for variation of the red sorghum extract MR-Buster from Cracka and Liberty included small sugars, flavones, flavanones, flavonols (and their glycosylated counterparts), unsaturated fatty acids, and several large un-resolved polyphenolic polymers. These putative identifications include commonly detected sorghum polyphenols, like apigenin, naringenin, luteolin, and eriodictyol. Both MR-Buster and Cracka were found to have large peaks at the higher end of the spectrum, most notably at *m/z* 689, 851, 1107, and 1269 (Supplementary Figure S9). Smaller peaks were found to surround these and were found to be separated by 16 Da (loss of hydroxyl group), while the larger separations included 162 (loss of sugar), and 272/255 Da (possible loss of flavonoid). These peaks are notably absent from Liberty extract. Cracka sorghum extract was found to have several overlapping mass bins to MR-Buster but with less diversity of metabolites as most mass bin identifications were of routinely identified sorghum polyphenols, including apigenin and naringenin. White sorghum (Liberty) extract presented little similarity to both red varieties as its mass bins contributing to variation included disaccharides, tricarboxylic acids, fatty acids, and lignans. Interestingly, the most abundant mass bins across the three sorghum extracts were essentially identical. The putative identifications made indicated the presence of phenylpropanoid glycerides, flavone/flavanones, disaccharides, and most predominately fatty acids, including oleic acid, linoleic acid, and oleic/linoleic acid-related compounds. These identified compounds, as well as the 10 most abundant from each extract, were then mapped to specific biosynthetic pathways using MetaboAnalyst Pathway Analysis (Figure 5).

## Discussion

While modern monogastric animal feed has been formulated for optimum nutrient utilization and digestive efficiency, performance gains still remain, especially in feeds composed of sorghum grain. With poultry production as a model, increases in efficiency with sorghum as a feed grain, as measured by FCR, have begun to level out (Supplementary Figure S1). While efforts to reduce anti-nutrient content in sorghum, most notably tannin and polyphenol concentrations, have been successful, gaps in efficiency and efficacy of feed additives remain, possibly due to unintended consequences in feed quality sorghum breeding. An understudied alternative analytical approach was thus used to identify anti-nutrients that may be causing varied performance in sorghum feed and to determine the suitability of different analytical techniques for assessing metabolic variation among sorghum grain extracts.

In this study, all three sorghum extracts, especially the Liberty variety, were found to have high ion counts for mass bins putatively identified as fatty acids, including oleic acid, linoleic acid, vernolic acid, ricinoleic acid, and trihydroxyoctadecenoic acid, which could result in nutritional variation in monogastric diets composed primarily of sorghum. With oleic acid as a starting point, vernolic acid is formed through an epoxidation reaction, ricinoleic acid through hydroxylation, linoleic acid through desaturation, and trihydroxyoctadecenoic acid through the hydroxylation of linoleic acid (Mazur et al., 1999; Cao and Zhang, 2013). The identifications made through mass spectrometry echo the strong, sharp FT-IR peaks between 3,000 and 2,900 cm^−1^ which correlate with C – H bonding found extensively in fatty acids (Shapaval et al., 2014). MR-Buster has been previously found to contain linoleic and oleic acids as the most dominant fatty acids, making up 80% of unsaturated fatty acid content (Mehmood et al., 2008). White varieties are similarly dominated by oleic and linoleic acids but at slightly higher proportions (Afify et al., 2012). Broiler chickens fed diets high in oleic acid were found to have a higher FCR, as well as reduced muscle and carcass weights (Toomer et al., 2003). Similar long chain fatty acids have also been shown to inhibit enzyme activity which could result in muted responses of exogenous feed enzymes (Kido et al., 1984). The high levels of fatty acids detected in the current study indicate the potential of implementing an exogenous lipase or emulsifier, as high levels of these compounds could be detrimental to growth and performance parameters.

Sorghum breeding efforts may have triggered metabolic alterations by favoring fatty acid synthesis over polyphenols, particularly condensed tannins. This hypothesis is supported by current work on sorghum grain and its management. Xie et al. (2019) studied sorghum varieties and preference by birds for feeding. They found that varieties avoided by birds had higher anthocyanin and tannin precursors (flavan-3-ols) than those that they preferred to eat. This correlated with the presence or absence of the *Tannin1* gene, previously found in sorghum to be involved in the regulation of polyphenols and tannins (Wu et al., 2012). In addition to determining the difference in polyphenols, the bird-preferred sorghum was found to have increased volatiles associated with fatty acids, as well as higher concentrations of fatty acids, including linolenic acid. Xie et al. (2019) concluded that the modulation of the *Tannin1* gene affects *SbGL2* which is involved in transcription of fatty acids.

In addition to the fatty acids and common polyphenols detected, the red sorghum varieties were found to have peaks with high ion masses and ion count, *m/z* 689, 851, 1107, and 1269, not clearly identifiable as traditional sorghum tannins. Similar peak masses have been previously identified in sorghum extracts as either pyrano-compounds or glucosylated heteropolyflavans. In sorghum leaf sheath, Khalil et al. (2010) describes the structural determination of a novel pyrano-3-deoxyanthocyanidin, pyrano-apigenindin [*m/z* 371.091 (+)]. Red and black sorghums were found to have unique flavanone structures, including pyrano-naringenin-catechin (*m/z* 689), pyrano-naringenin-catechin-glucoside (*m/z* 851), pyrano-eriodictyol-catechin-glucoside (*m/z* 867), and pyrano-naringenin-pyrano-eriodictyol-catechin (*m/z* 1107) (Yang, 2013; Rao et al., 2018). Glucosylated heteropolyflavans, described by Gujer et al. (1986) and Krueger et al. (2003), are composed of unique polymerizations of eriodictyol/naringenin and luteolinidin/apigeninidin with varying degrees of glycosylation. The masses of these compounds were predicted with the equation 288 + 272*a* + 256*b* + 162*c* + cation with 288 representing the mass of an eriodictyol base unit, 272 referring to a proluteolinidin unit, 256 to a proapigeninidin unit, 162 to additional sugar units, and the letters referring to possible degrees of polymerization. The MS^2^ spectra support these identifications as the primary fragment masses detected correspond to losses of 256, 272, and 162. Purification of these unknowns is needed along with structural evidence that could be gained using NMR techniques.

In the current study, spectra obtained from ESI (+, −) were clearer than those produced using MALDI (+) with a greater number of clearly identifiable peaks, most likely due to the lack of need for a matrix compound with ESI. ESI (+, −) analyses were also successful in detecting routinely identified polyphenol compounds in sorghum, including apigenin, naringenin, and phenylpropanoid glycerides (Kang et al., 2016). Sorghum grain extracts have been sparingly analyzed using MALDI (Krueger et al., 2003; Qi et al., 2018; Jiang et al., 2020; Reeves et al., 2020). Unsupervised analysis of the ESI and MALDI spectra allowed for the clear separation between red and white varieties and in some cases among all three extracts. Identifications allowed for pathways of interest to be highlighted, as well as comparisons of percent relative abundances of selected masses. These metabolomic methods are most likely critical when first releasing a new variety of grain to the market. Recently, Zhou et al. (2020) compared three sorghum varieties (black, red, and white) to study metabolic variation based on color. PCA revealed a separation of grains based on grain color, and compound identification found that darker grains contained more flavonoids than lighter colored grains, similar to what was determined in the current work. Typically, the color of the grain gives some indication of the polyphenols present with darker and highly colored grains containing higher concentrations and often larger, more complex polyphenols (Rhodes et al., 2014).

FT-IR has been previously shown to be successful in both distinguishing bulk differences among general metabolite profiles (Johnson et al., 2003) as well as nuancing more subtle variations, e.g., tannin extracts separated based on tannin chemistry (Grasel et al., 2016a). In the current study, FT-IR analysis revealed there were structural similarities, with regard to polyphenols and tannins, among the sorghum polyphenol extracts. The subtle differences in sorghum extract peak structures and maxima suggest the presence of competing compounds indicative of a complex plant extract. As reviewed by Ricci et al. (2015), the spectra matched the general profile of extracts containing phenolic, polyphenolic, and tannin compounds, including characteristic O – H hydroxyl groups, aromatic C – H bonds, aromatic C = C bonds, and C – O groups. A similar approach in evaluating the presence/absence of specific metabolite structures was taken by Cameron et al. (2006) in their investigation into alteration to lignin and suberin content in grasses, legumes, and forbs subject to attack by a root hemiparasitic plant.

Comparable values for the O – H maxima have previously been reported in sorghum flour (Manuhara et al., 2017). The strong bands around 3,000 cm^−1^, indicating aliphatic C – H structures, have been detected in similar extract types, including sorghum, and possibly indicate the presence of sugars and/or fatty acids (She et al., 2010; Shapaval et al., 2014; Manuhara et al., 2017). In their natural state, polyphenols are most likely to be conjugated to sugars (Bravo, 1998). Polyphenol and tannin extracts typically contain peaks corresponding to C = O groups (1,700 s cm^−1^), especially those containing hydrolyzable tannins (Grasel et al., 2016b; Reeves et al., 2020). This region can also indicate the presence of amide functional groups, most commonly found in proteins. Duodu et al. (2001) studied protein structure in highly digestible sorghum and maize mutants and identified bands between 1,670 and 1,620 cm^−1^ as amide I and from 1,550 to 1,500 cm^−1^ as amide II. The subtle differences between red and white sorghum grains in these regions may indicate important differences in protein content and structure with possible nutritional implications. Selle et al. (2020) studied amino acids and kafirin protein in several sorghum varieties, including a Buster variety and Liberty. With regard to crude protein and kafirin content, Liberty had 80.9 and 41.4 g/kg, while Buster reported 99.2 and 44.6 g/kg, respectively (Selle et al., 2020). The higher proportion of kafirin protein found in Liberty may be causing the spectral differences observed in the current study.

The region from 1,630 to 1,400 cm^−1^ is strongly diagnostic for the presence of polyphenols as it indicates aromatic C = C bonding found in the aromatic rings of phenolic compounds. The peaks identified between 1,400 and 1,000 cm^−1^ are also characteristic of C – OH as well as C – O – C bonding (Ricci et al., 2015). The differences in the sorghum extract spectra in these regions indicate that the white variety (Liberty) likely has reduced polyphenol content compared to the red varieties (MR-Buster, Cracka), a common finding in sorghum polyphenol studies, which can correlate with nutritional variations observed in feeding (Truong et al., 2016). FT-IR is a useful, simple method of analysis that could be used in on-line agricultural settings, as opposed to full scale laboratories. Although this study analyzed more laboratory intensive freeze-dried extracts, FT-IR can easily be applied to simple liquid extracts taken on-site.

This study of an alternate analytical framework for polyphenol characterization highlighted the need for complementary methods to fully understand the complexity of sorghum polyphenol extracts. FT-IR spectroscopy provided general chemical profiles which highlighted functional groups and classes of compounds specific to polyphenol and tannin structural chemistry. Multivariate analysis of the FT-IR spectra demonstrated that the technique was robust enough to separate different extract types and to explain greater variance in the data than any MS method. Both ESI analyses produced similar plots to that of FT-IR, albeit with slightly better grouping of the sorghum extracts. ESI also provided a clearer metabolite profile than MALDI. These results indicated that, with regard to untargeted analysis, FT-IR and ESI provide essentially the same end-product allowing for similar conclusions to be drawn on bulk differences in the spectra. Based on these results, compatibility, and pricing, FT-IR may be the most effective tool for determining the applicability of certain grains to feed formulations, particularly with regard to polyphenol content. This application could be especially important in varietal selection for grain breeding and feed applications. Markers for chosen nutritional parameters, such as protein structure and anti-nutrient content, could be selected for and used as a screening tool prior to more intensive analytical methodologies should they be needed. However, mass spectrometric studies of metabolites present in these grains should be used to guide the interpretation of FT-IR spectra in the field to further highlight subtle differences in the grains that may result in monogastric feed performance variation.

## Data Availability Statement

The original contributions presented in the study are included in the article/Supplementary Material, further inquiries can be directed to the corresponding author.

## Author Contributions

All authors contributed to the conception and design of the study. HH collected the data. HH, RF, and DC analyzed the data. HH wrote the manuscript. All authors contributed to manuscript revision, reading, and approval of the submitted version.

### Conflict of Interest

AC is employed by DSM Nutritional Products.

The remaining authors declare that the research was conducted in the absence of any commercial or financial relationships that could be construed as a potential conflict of interest.

## Footnotes

^1^https://www.metaboanalyst.ca/
