# Supplementary material for: Latent Anti-nutrients and Unintentional Breeding Consequences in Australian Sorghum bicolor Varieties
Source: Front Plant Sci. 2021 Mar 1;12:625260. doi: 10.3389/fpls.2021.625260 (PMC7959176; doi:10.3389/fpls.2021.625260)
Supplement: Supplementary file 1 [file Presentation_1.pptx]

## Slide 1
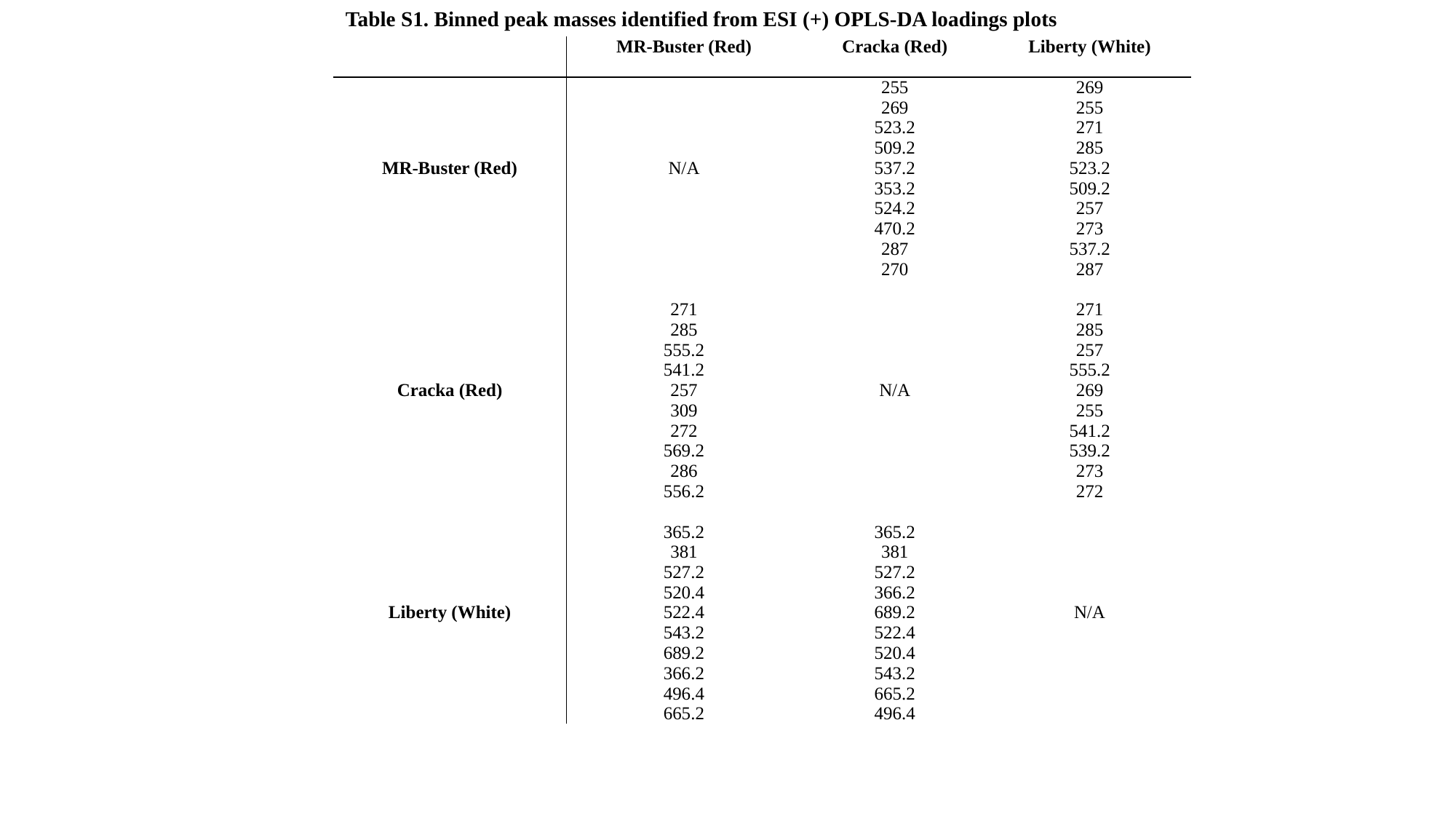

Table S1. Binned peak masses identified from ESI (+) OPLS-DA loadings plots
| | MR-Buster (Red) | Cracka (Red) | Liberty (White) |
| --- | --- | --- | --- |
| MR-Buster (Red) | N/A | 255 269 523.2 509.2 537.2 353.2 524.2 470.2 287 270 | 269 255 271 285 523.2 509.2 257 273 537.2 287 |
| Cracka (Red) | 271 285 555.2 541.2 257 309 272 569.2 286 556.2 | N/A | 271 285 257 555.2 269 255 541.2 539.2 273 272 |
| Liberty (White) | 365.2 381 527.2 520.4 522.4 543.2 689.2 366.2 496.4 665.2 | 365.2 381 527.2 366.2 689.2 522.4 520.4 543.2 665.2 496.4 | N/A |

## Slide 2
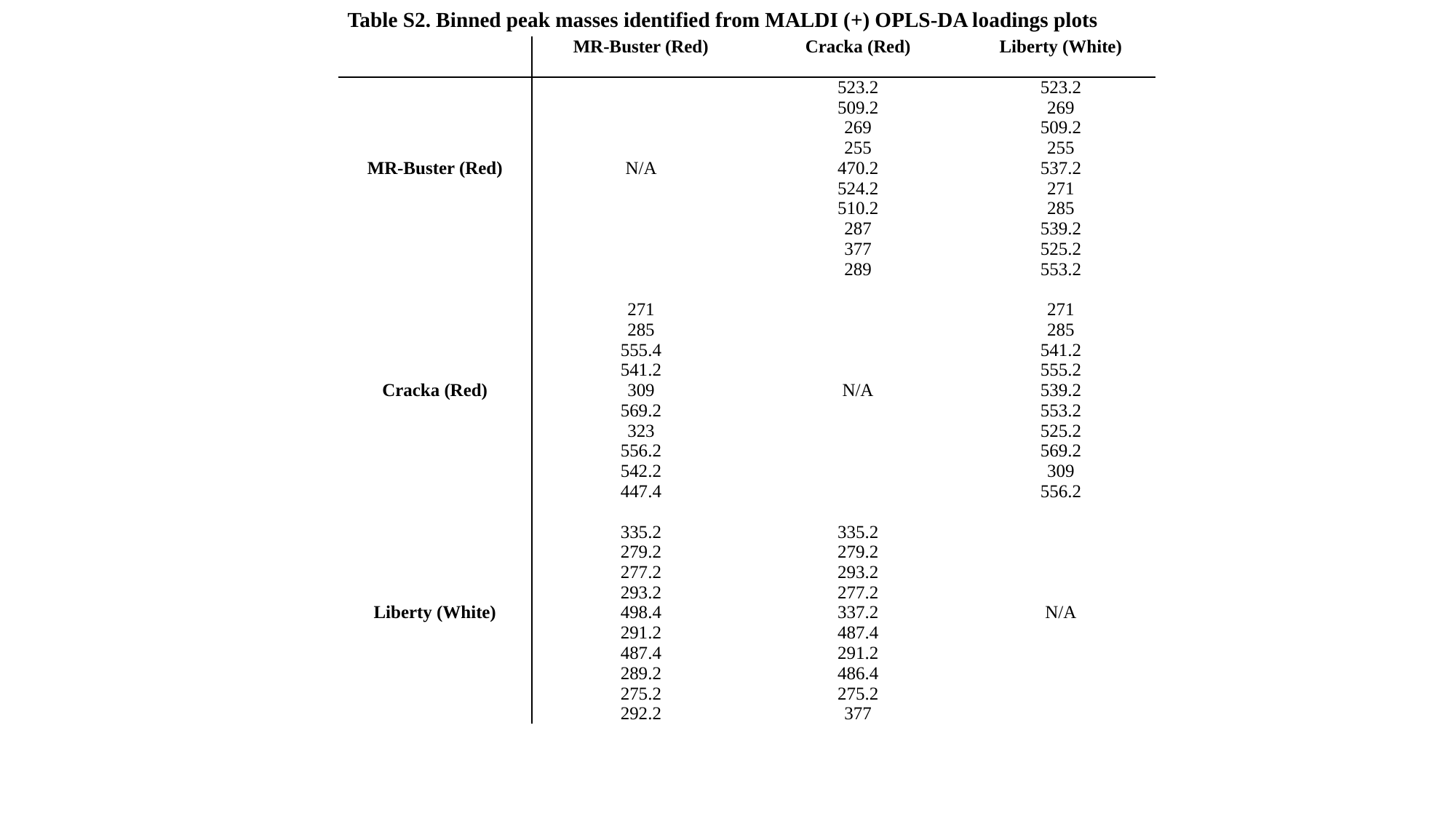

Table S2. Binned peak masses identified from MALDI (+) OPLS-DA loadings plots
| | MR-Buster (Red) | Cracka (Red) | Liberty (White) |
| --- | --- | --- | --- |
| MR-Buster (Red) | N/A | 523.2 509.2 269 255 470.2 524.2 510.2 287 377 289 | 523.2 269 509.2 255 537.2 271 285 539.2 525.2 553.2 |
| Cracka (Red) | 271 285 555.4 541.2 309 569.2 323 556.2 542.2 447.4 | N/A | 271 285 541.2 555.2 539.2 553.2 525.2 569.2 309 556.2 |
| Liberty (White) | 335.2 279.2 277.2 293.2 498.4 291.2 487.4 289.2 275.2 292.2 | 335.2 279.2 293.2 277.2 337.2 487.4 291.2 486.4 275.2 377 | N/A |

## Slide 3
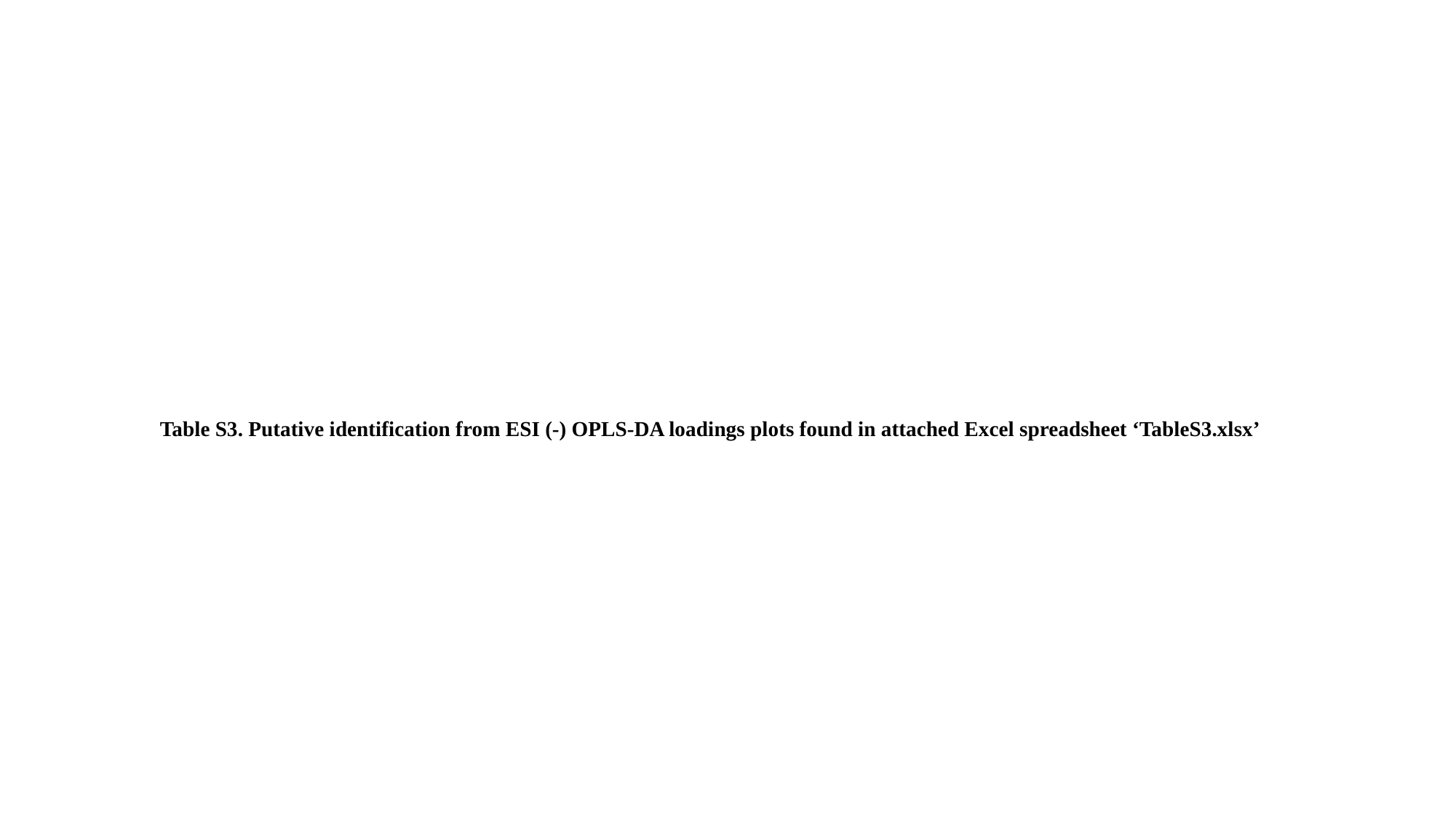

Table S3. Putative identification from ESI (-) OPLS-DA loadings plots found in attached Excel spreadsheet ‘TableS3.xlsx’

## Slide 4
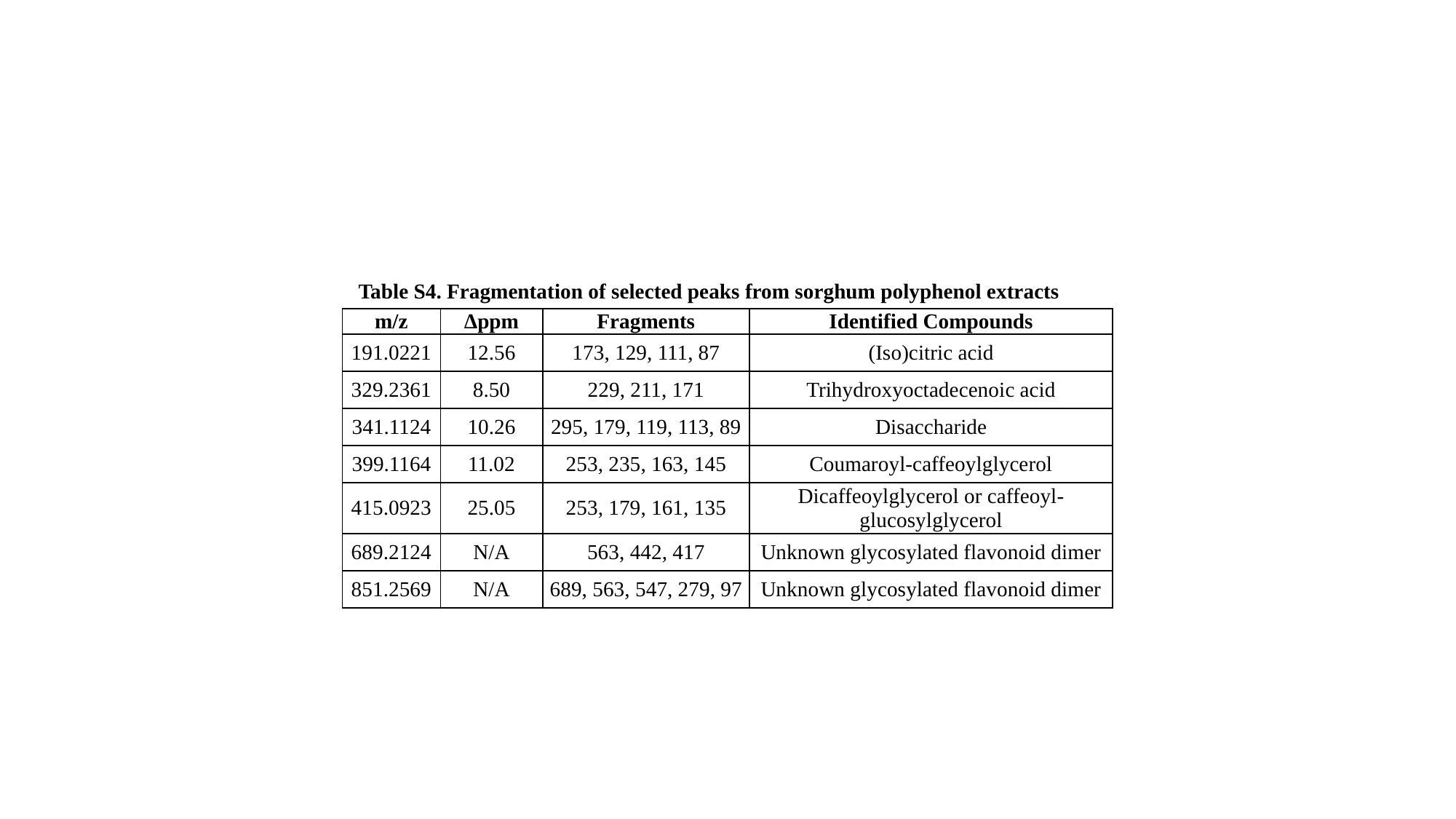

Table S4. Fragmentation of selected peaks from sorghum polyphenol extracts
| m/z | ∆ppm | Fragments | Identified Compounds |
| --- | --- | --- | --- |
| 191.0221 | 12.56 | 173, 129, 111, 87 | (Iso)citric acid |
| 329.2361 | 8.50 | 229, 211, 171 | Trihydroxyoctadecenoic acid |
| 341.1124 | 10.26 | 295, 179, 119, 113, 89 | Disaccharide |
| 399.1164 | 11.02 | 253, 235, 163, 145 | Coumaroyl-caffeoylglycerol |
| 415.0923 | 25.05 | 253, 179, 161, 135 | Dicaffeoylglycerol or caffeoyl-glucosylglycerol |
| 689.2124 | N/A | 563, 442, 417 | Unknown glycosylated flavonoid dimer |
| 851.2569 | N/A | 689, 563, 547, 279, 97 | Unknown glycosylated flavonoid dimer |

## Slide 5
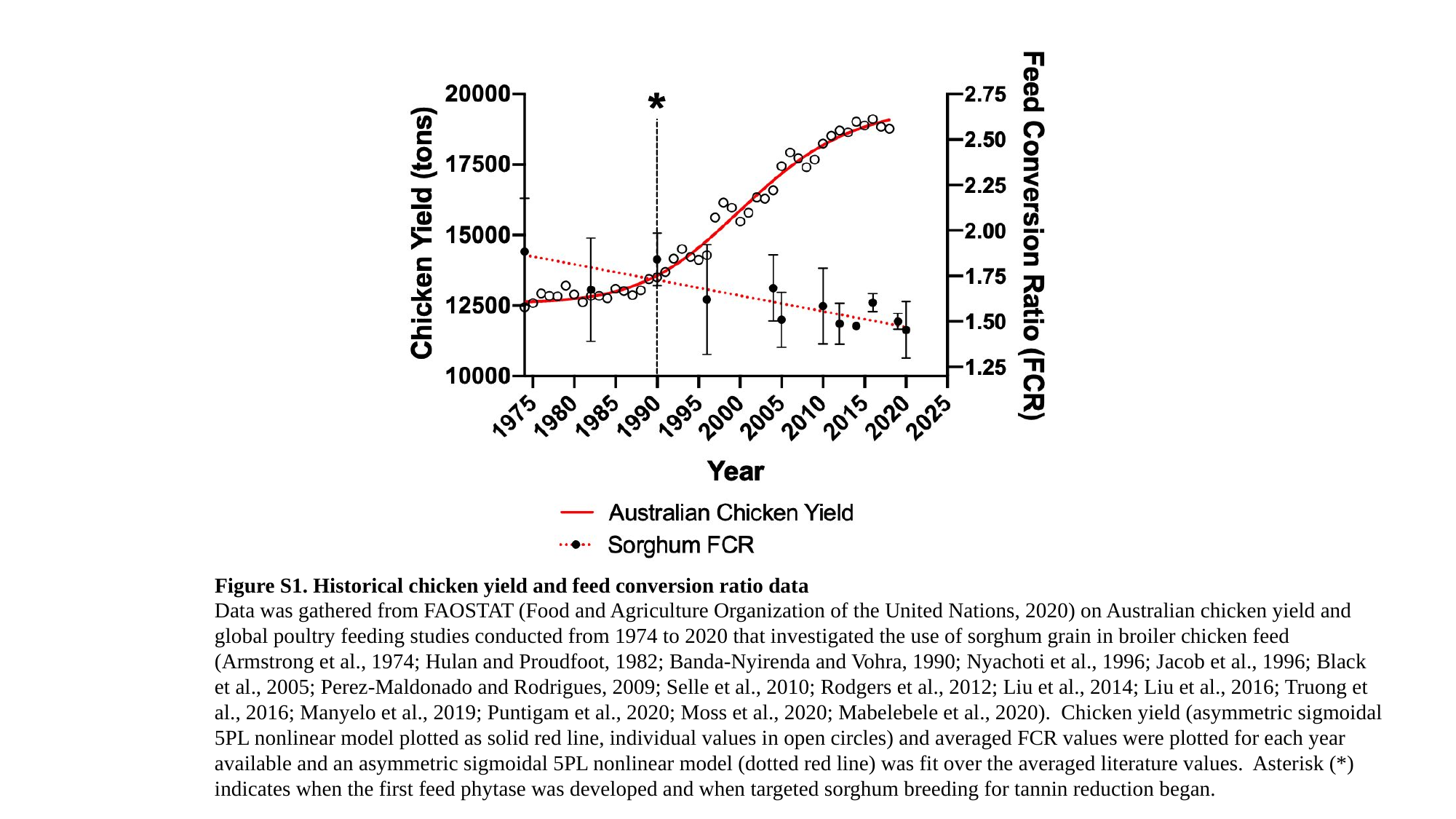

Figure S1. Historical chicken yield and feed conversion ratio data
Data was gathered from FAOSTAT (Food and Agriculture Organization of the United Nations, 2020) on Australian chicken yield and global poultry feeding studies conducted from 1974 to 2020 that investigated the use of sorghum grain in broiler chicken feed (Armstrong et al., 1974; Hulan and Proudfoot, 1982; Banda-Nyirenda and Vohra, 1990; Nyachoti et al., 1996; Jacob et al., 1996; Black et al., 2005; Perez-Maldonado and Rodrigues, 2009; Selle et al., 2010; Rodgers et al., 2012; Liu et al., 2014; Liu et al., 2016; Truong et al., 2016; Manyelo et al., 2019; Puntigam et al., 2020; Moss et al., 2020; Mabelebele et al., 2020). Chicken yield (asymmetric sigmoidal 5PL nonlinear model plotted as solid red line, individual values in open circles) and averaged FCR values were plotted for each year available and an asymmetric sigmoidal 5PL nonlinear model (dotted red line) was fit over the averaged literature values. Asterisk (*) indicates when the first feed phytase was developed and when targeted sorghum breeding for tannin reduction began.

## Slide 6
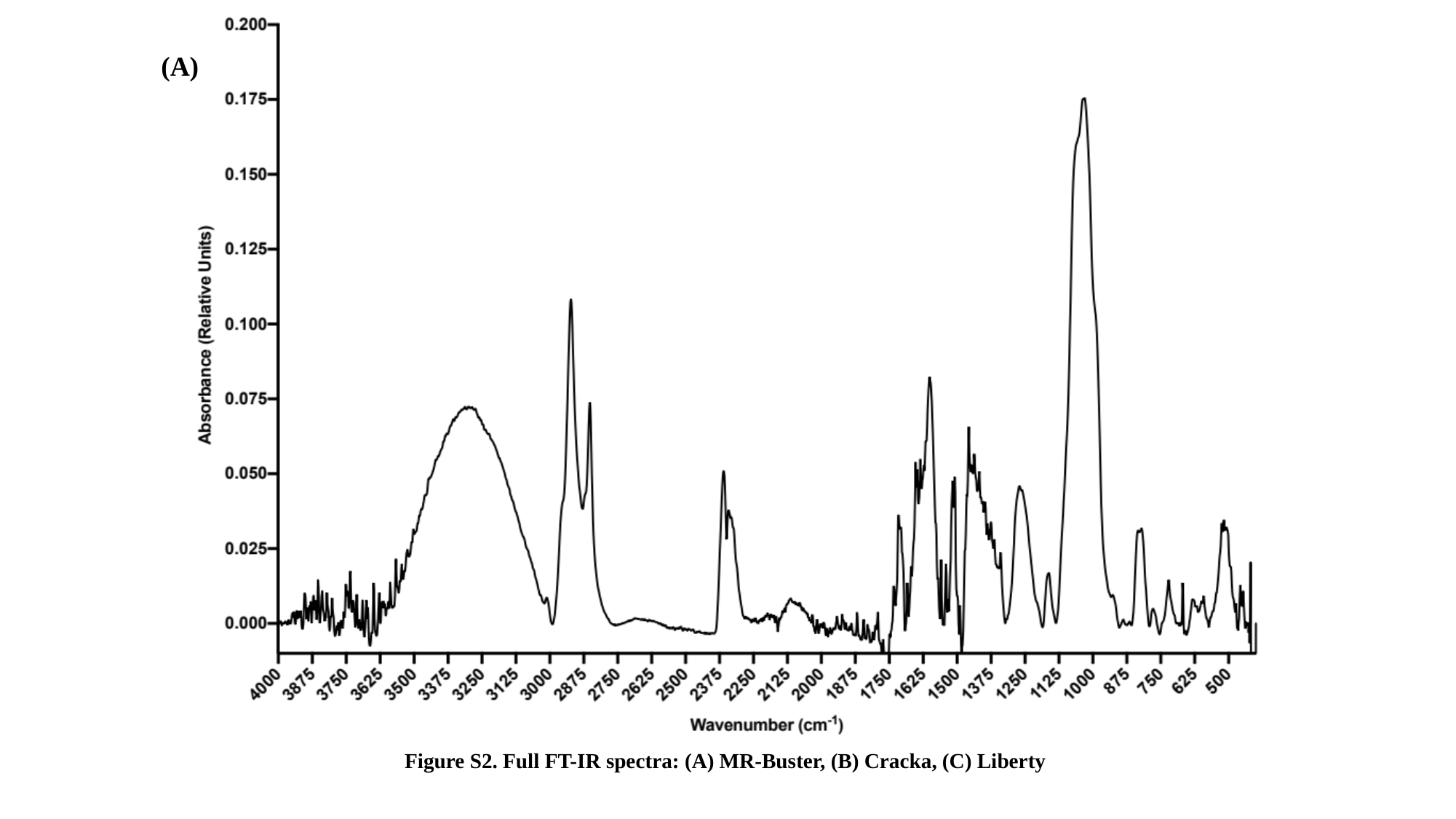

(A)
Figure S2. Full FT-IR spectra: (A) MR-Buster, (B) Cracka, (C) Liberty

## Slide 7
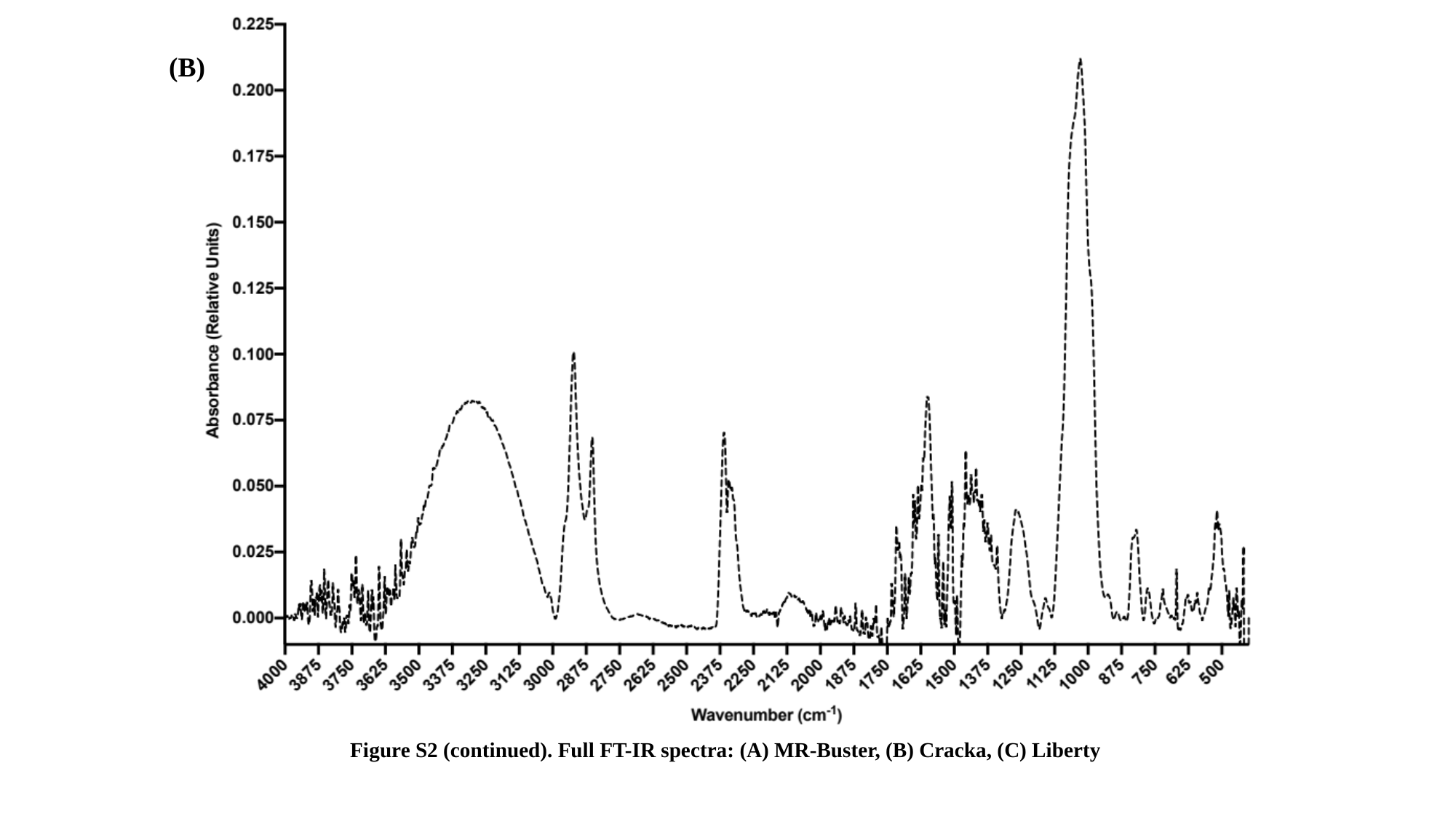

(B)
Figure S2 (continued). Full FT-IR spectra: (A) MR-Buster, (B) Cracka, (C) Liberty

## Slide 8
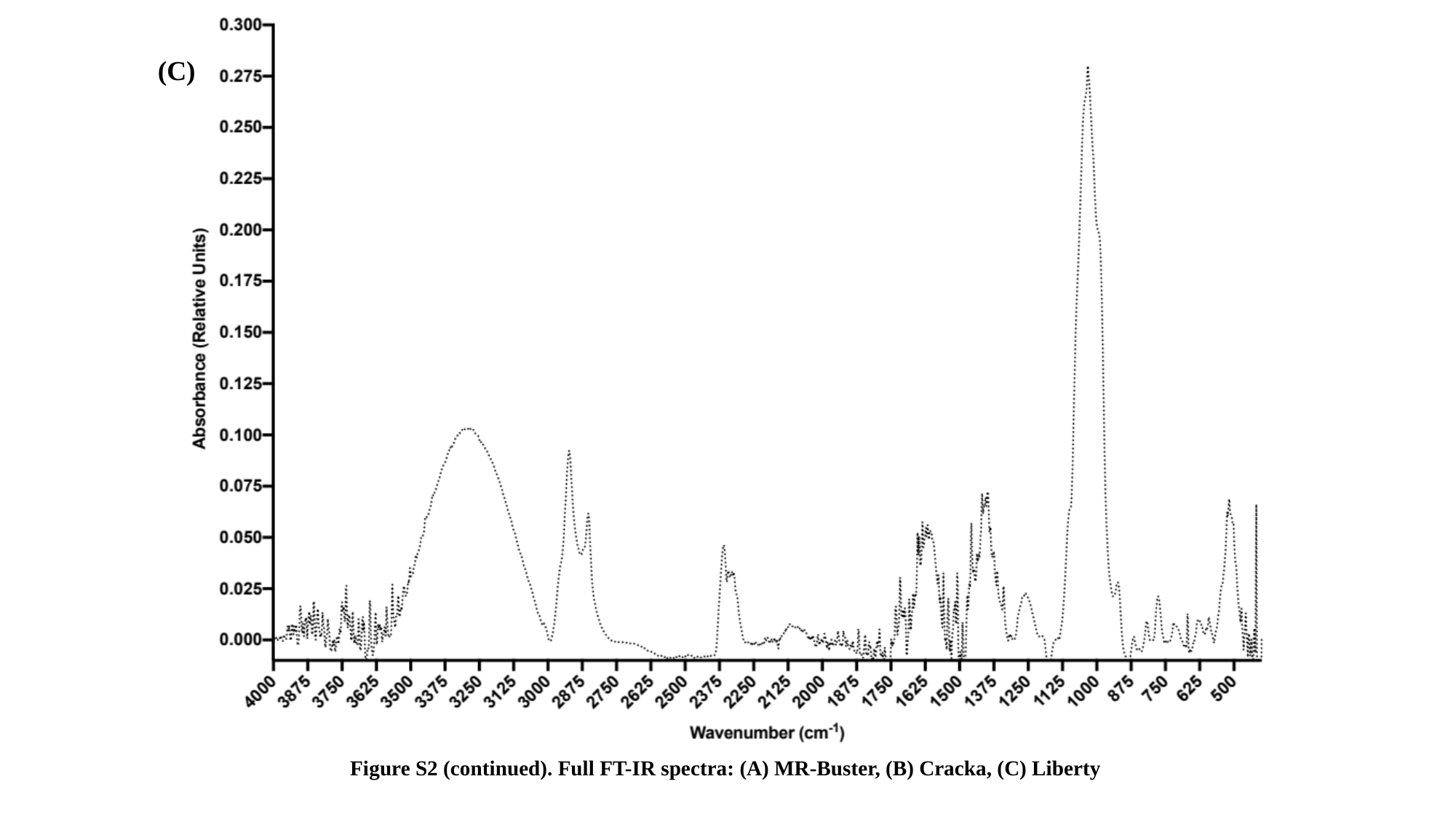

(C)
Figure S2 (continued). Full FT-IR spectra: (A) MR-Buster, (B) Cracka, (C) Liberty

## Slide 9
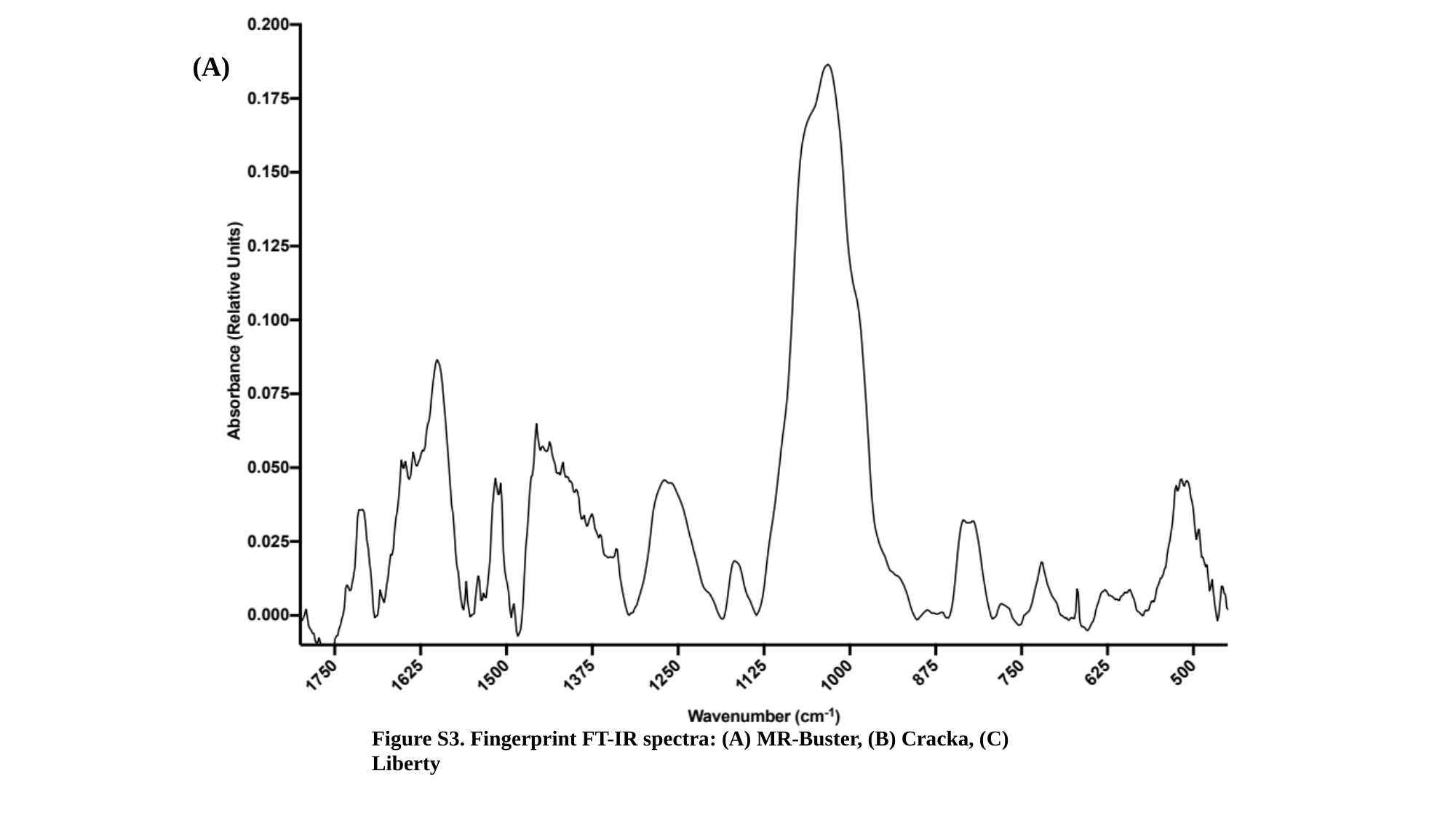

(A)
Figure S3. Fingerprint FT-IR spectra: (A) MR-Buster, (B) Cracka, (C) Liberty

## Slide 10
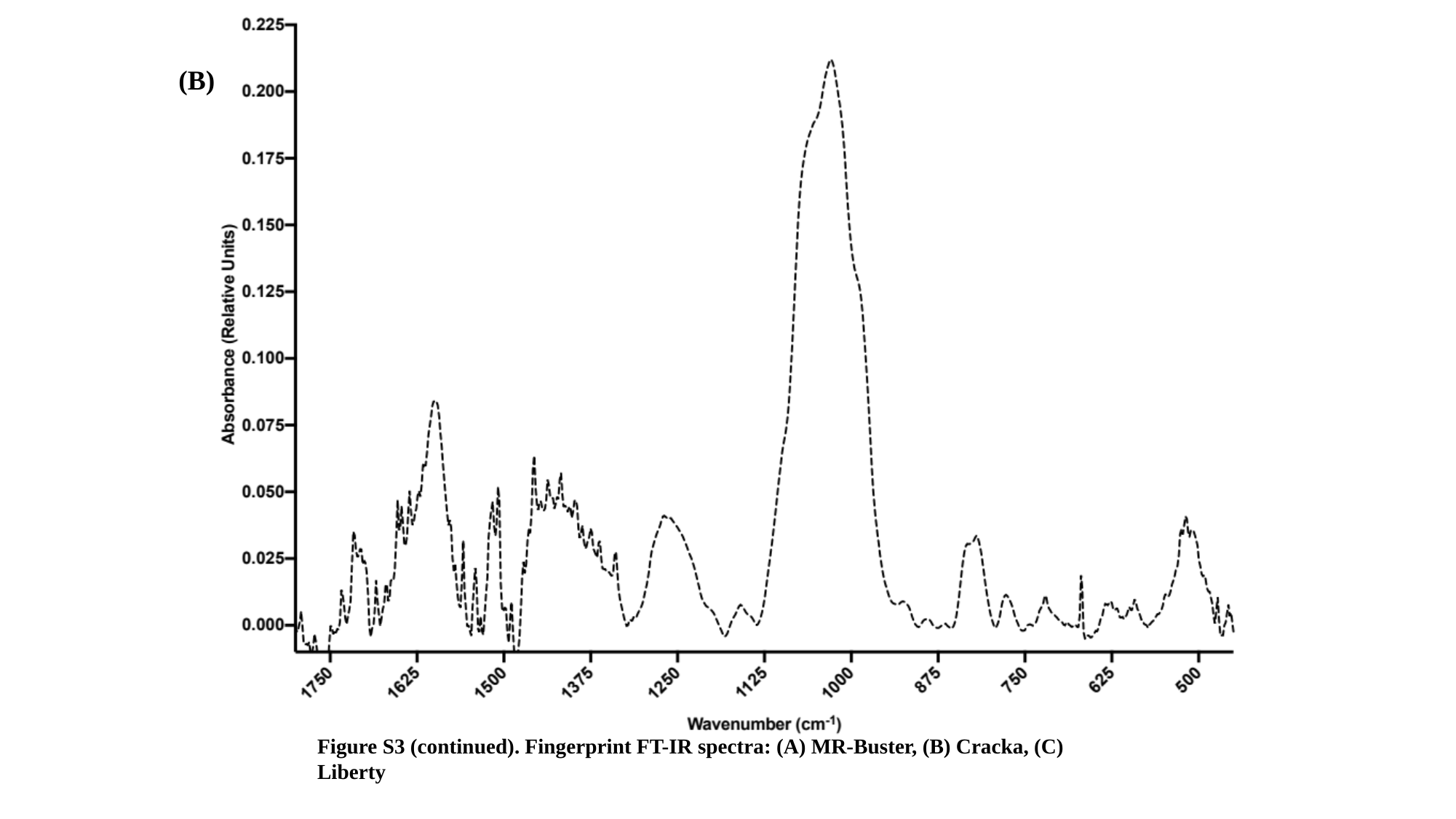

(B)
Figure S3 (continued). Fingerprint FT-IR spectra: (A) MR-Buster, (B) Cracka, (C) Liberty

## Slide 11
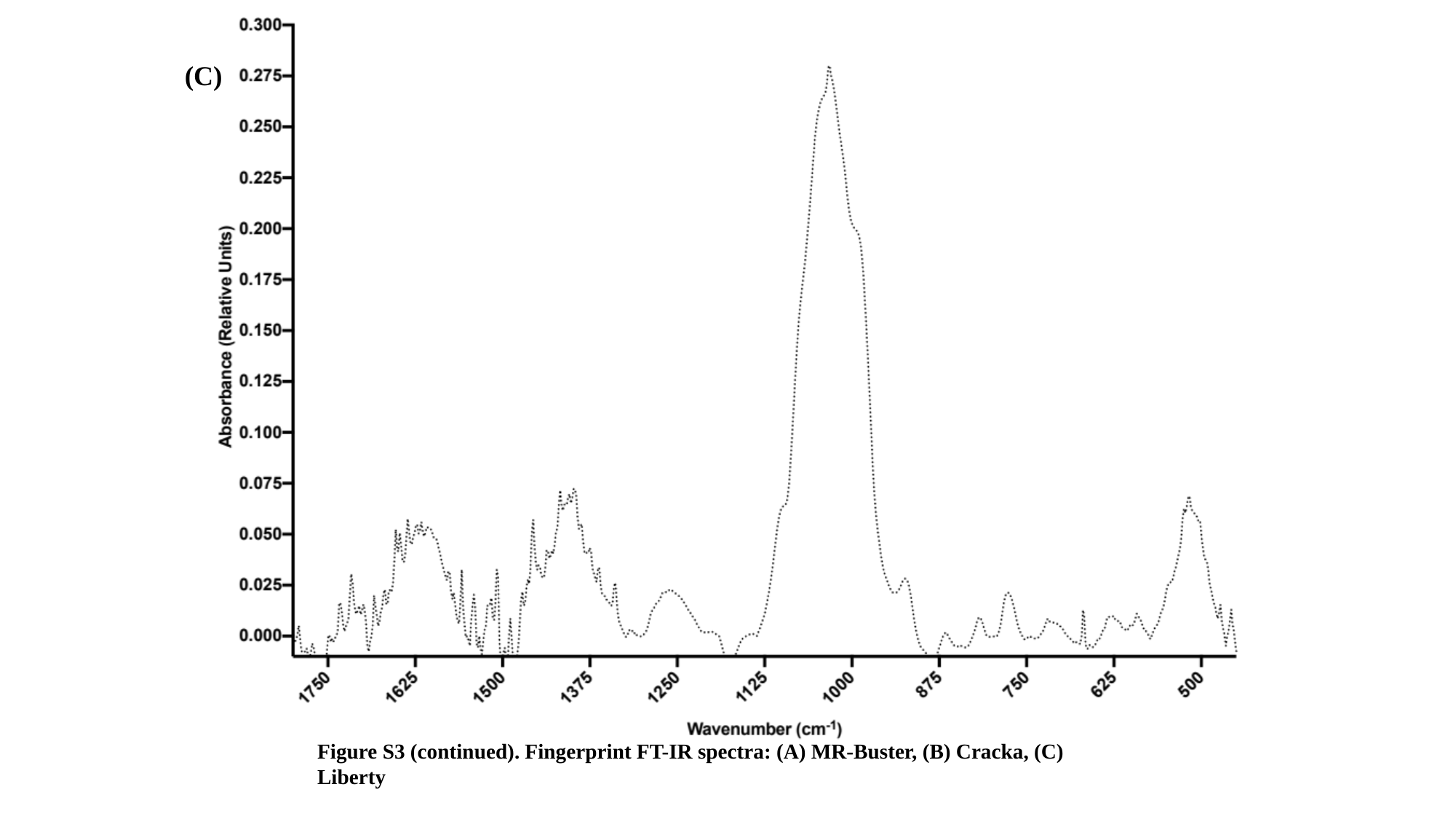

(C)
Figure S3 (continued). Fingerprint FT-IR spectra: (A) MR-Buster, (B) Cracka, (C) Liberty

## Slide 12
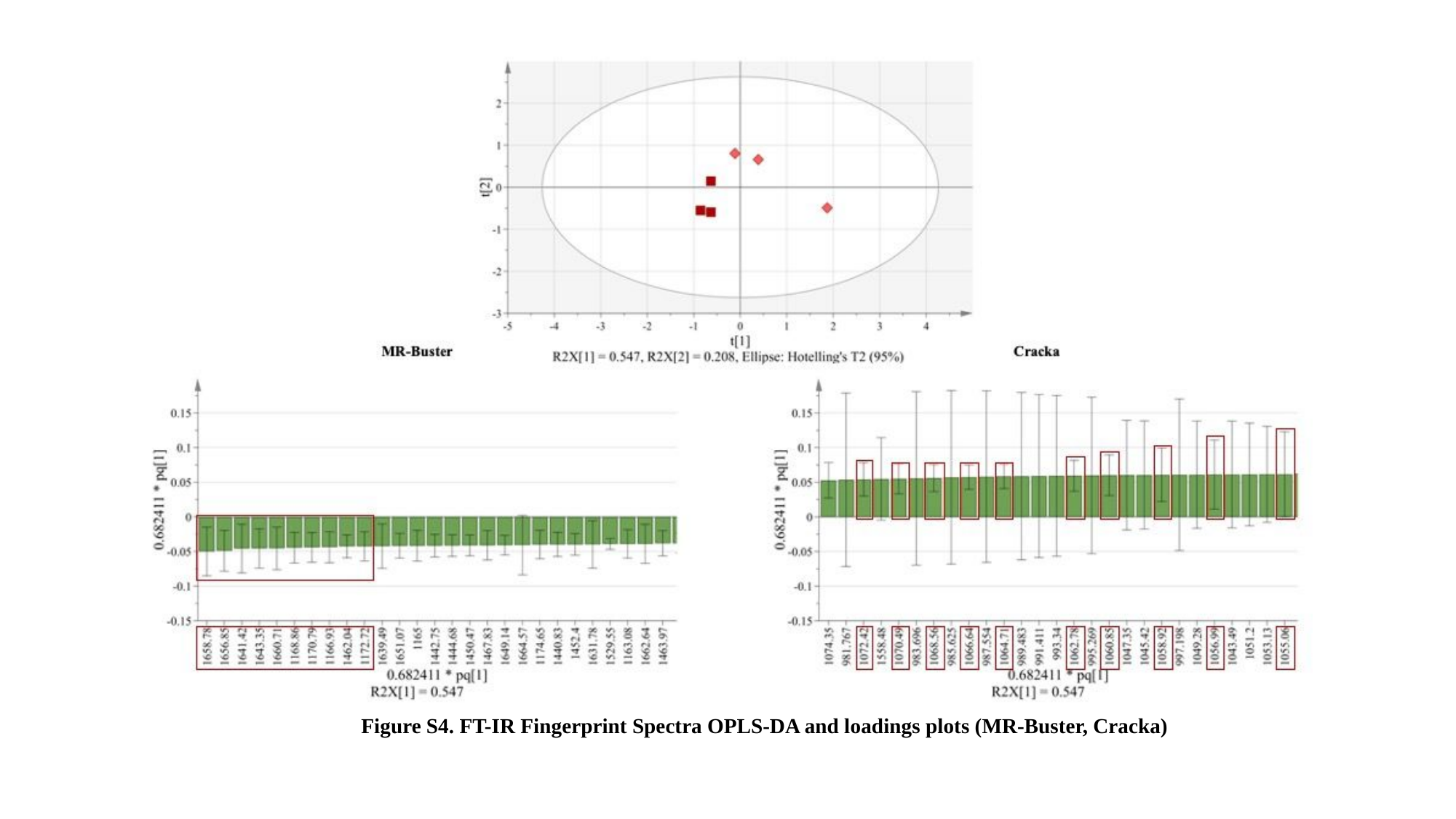

Figure S4. FT-IR Fingerprint Spectra OPLS-DA and loadings plots (MR-Buster, Cracka)

## Slide 13
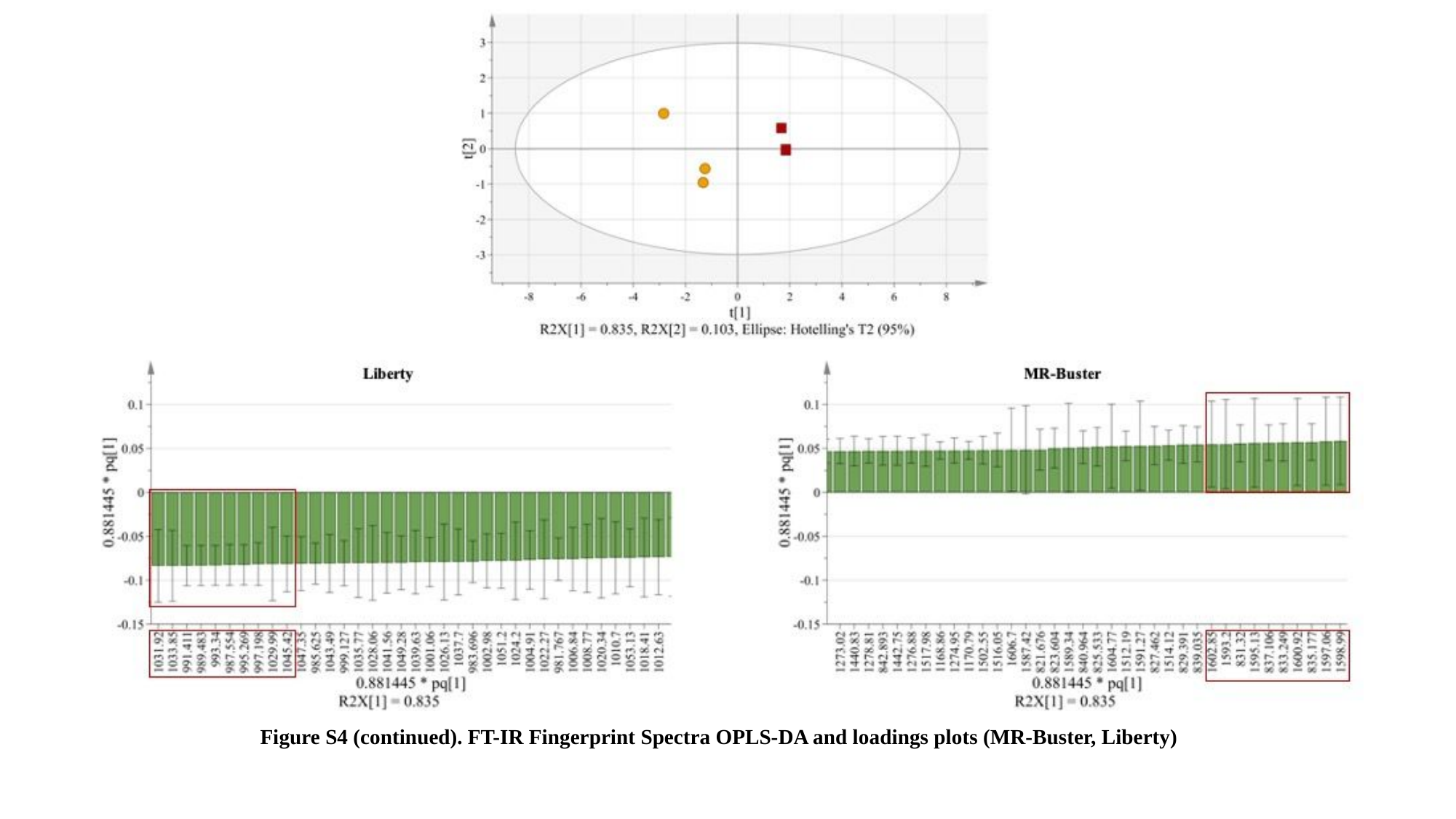

Figure S4 (continued). FT-IR Fingerprint Spectra OPLS-DA and loadings plots (MR-Buster, Liberty)

## Slide 14
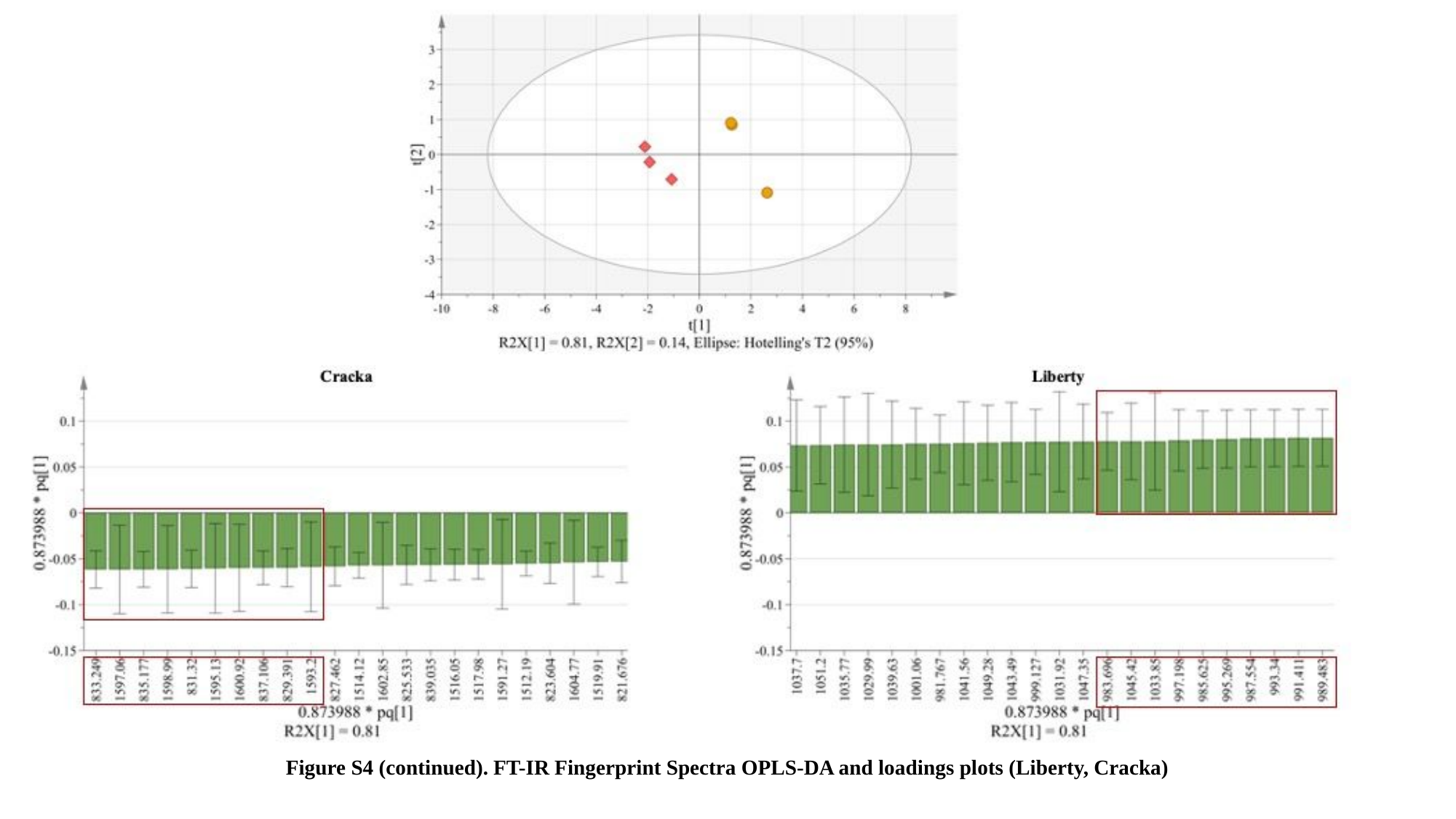

Figure S4 (continued). FT-IR Fingerprint Spectra OPLS-DA and loadings plots (Liberty, Cracka)

## Slide 15
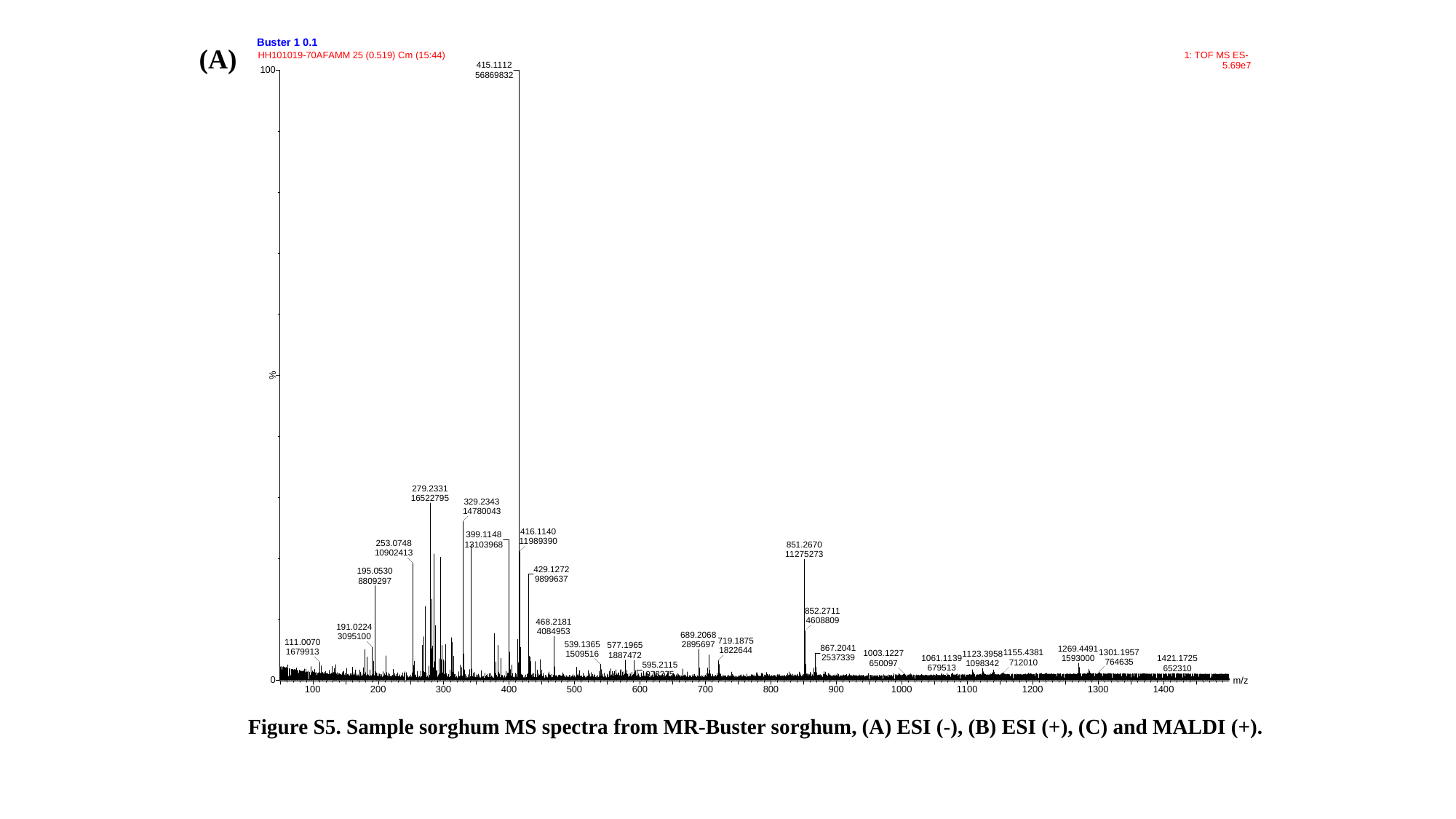

(A)
Figure S5. Sample sorghum MS spectra from MR-Buster sorghum, (A) ESI (-), (B) ESI (+), (C) and MALDI (+).

## Slide 16
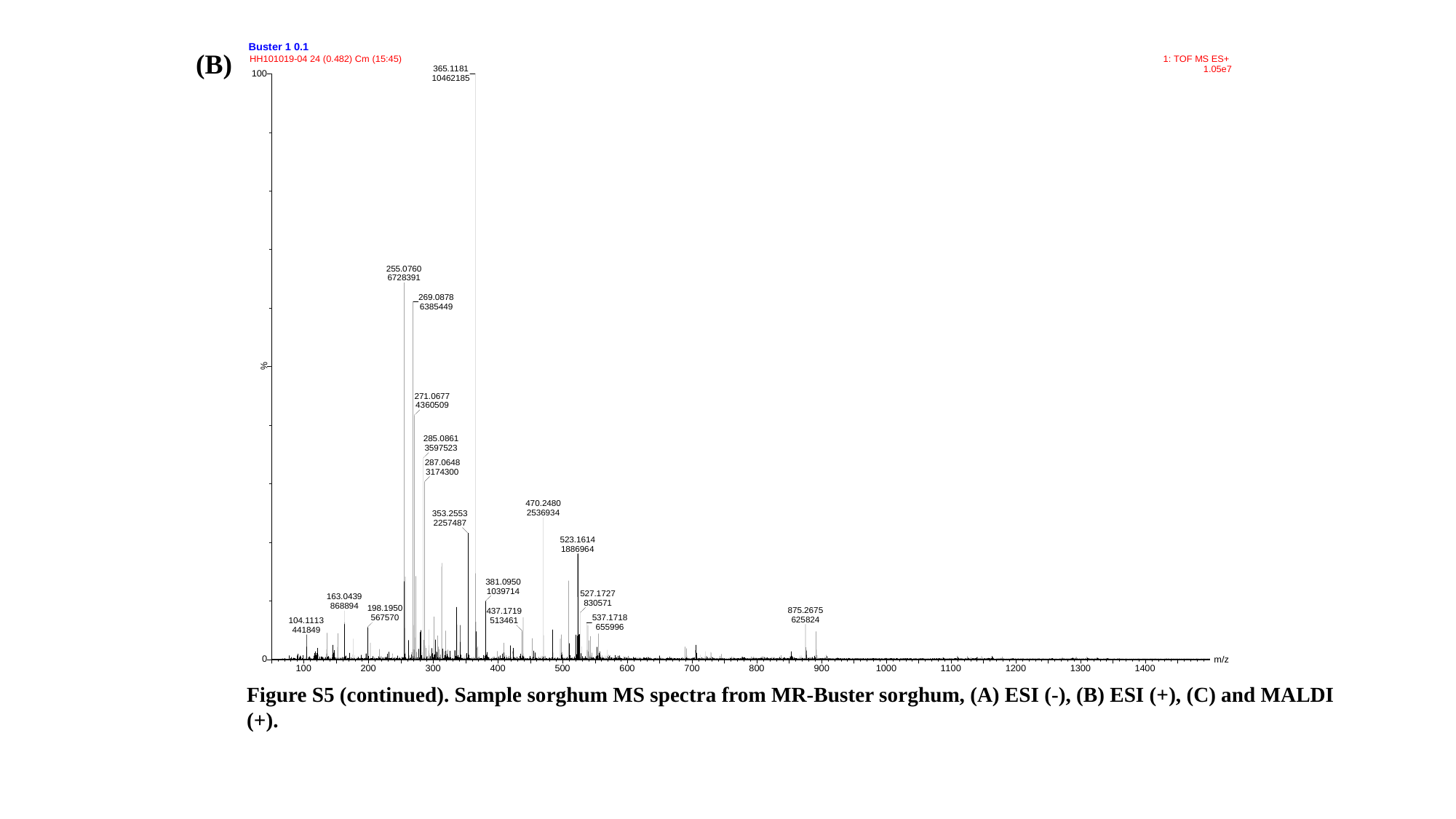

(B)
Figure S5 (continued). Sample sorghum MS spectra from MR-Buster sorghum, (A) ESI (-), (B) ESI (+), (C) and MALDI (+).

## Slide 17
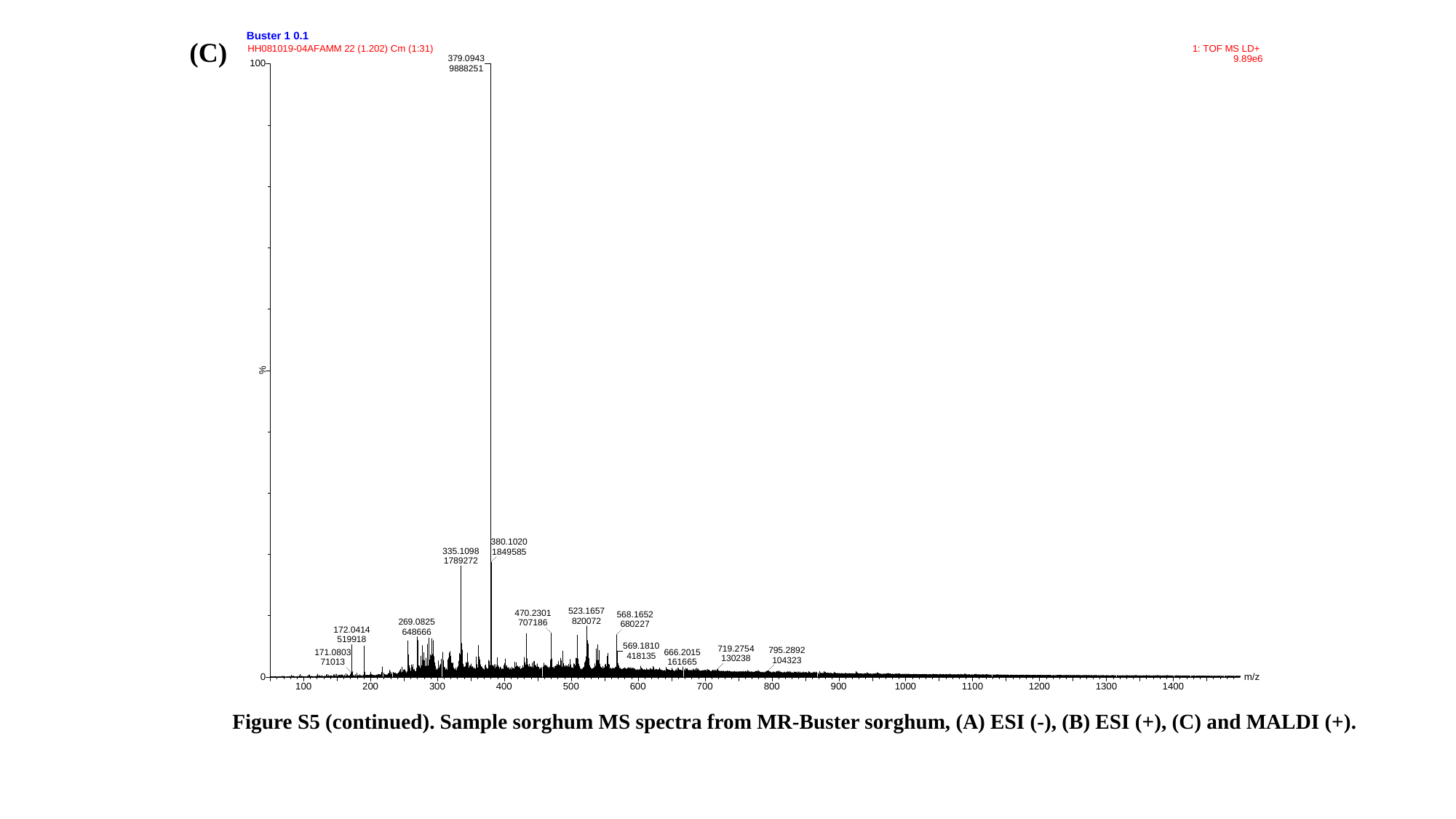

(C)
Figure S5 (continued). Sample sorghum MS spectra from MR-Buster sorghum, (A) ESI (-), (B) ESI (+), (C) and MALDI (+).

## Slide 18
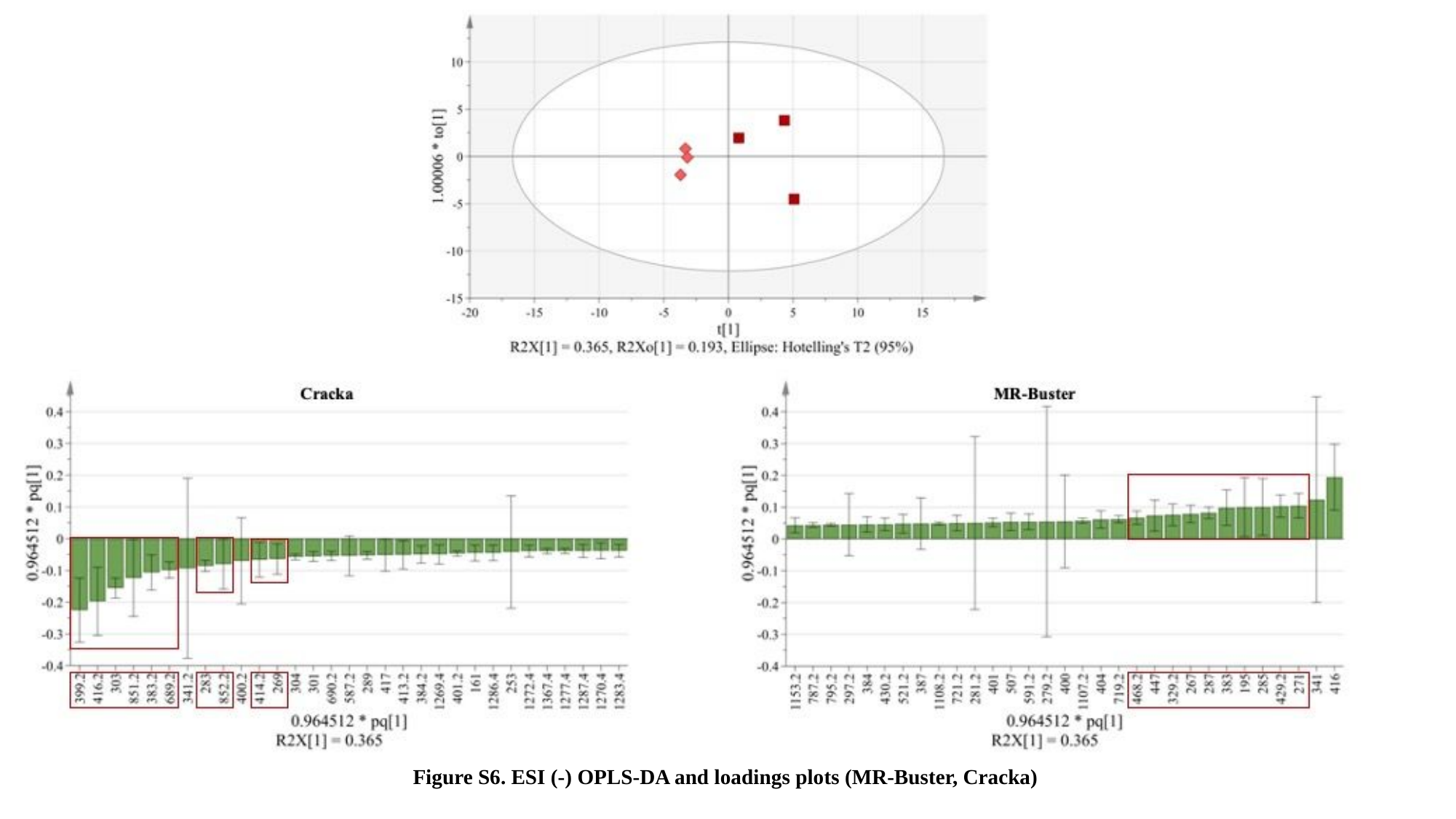

Figure S6. ESI (-) OPLS-DA and loadings plots (MR-Buster, Cracka)

## Slide 19
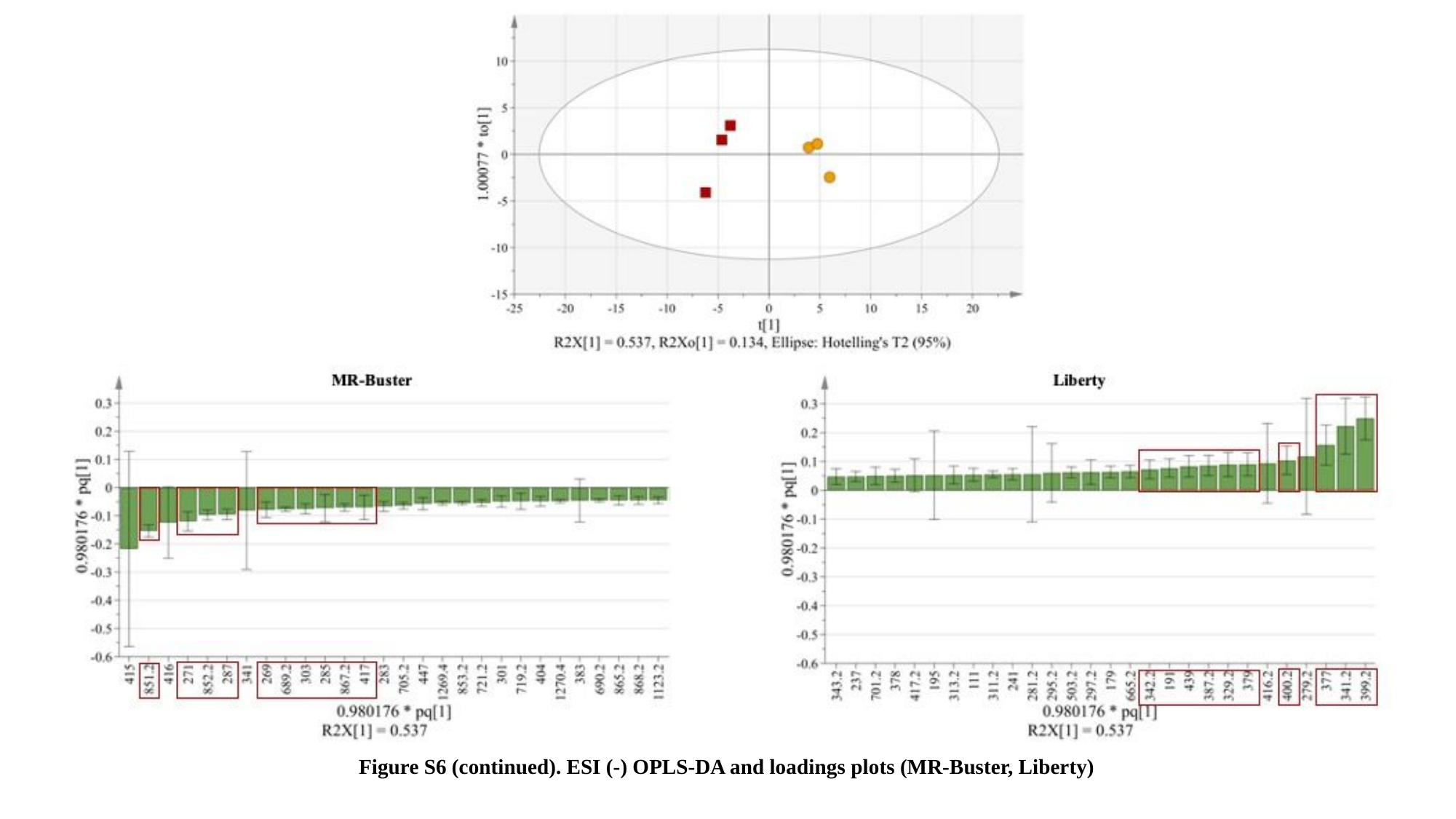

Figure S6 (continued). ESI (-) OPLS-DA and loadings plots (MR-Buster, Liberty)

## Slide 20
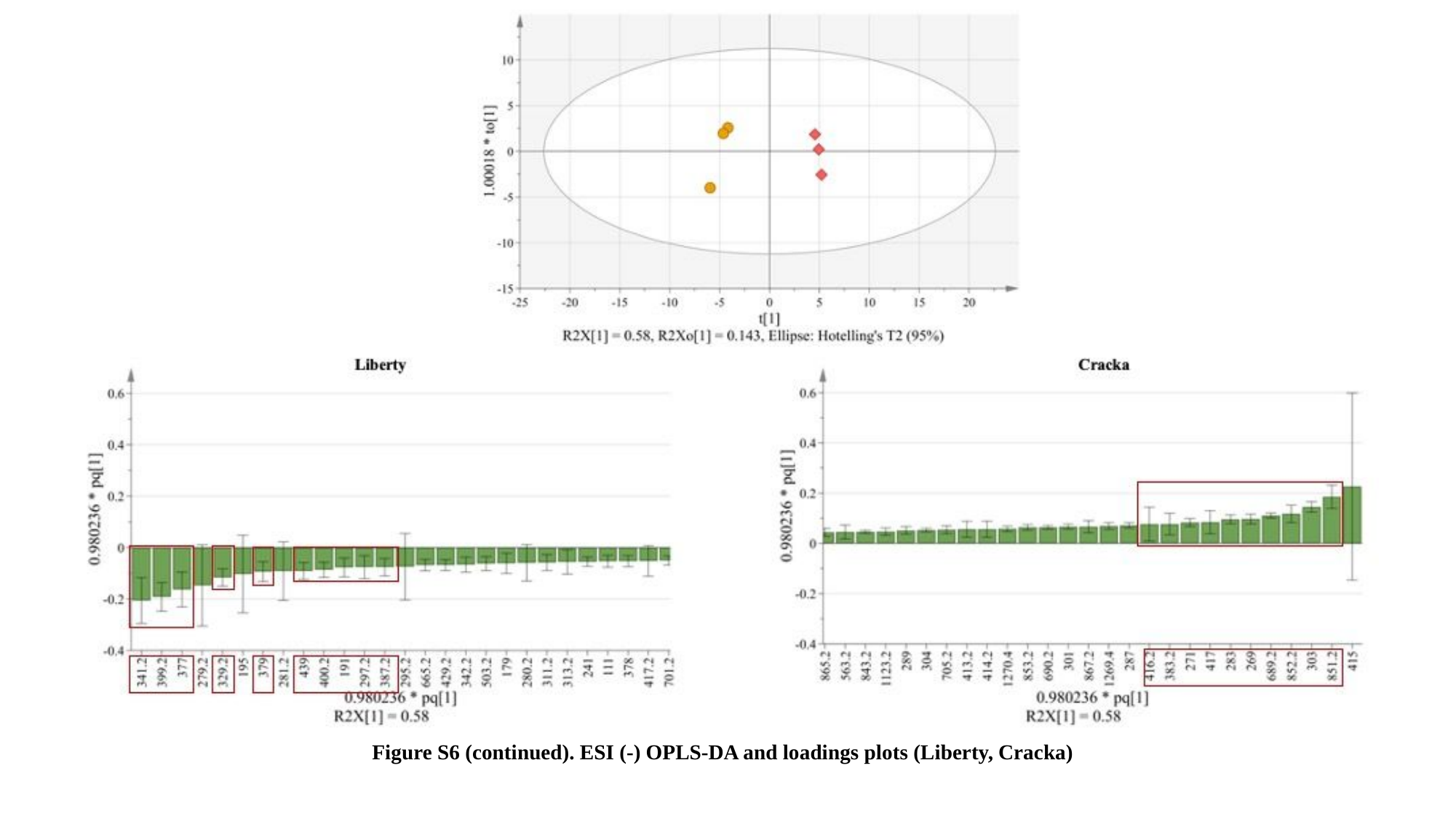

Figure S6 (continued). ESI (-) OPLS-DA and loadings plots (Liberty, Cracka)

## Slide 21
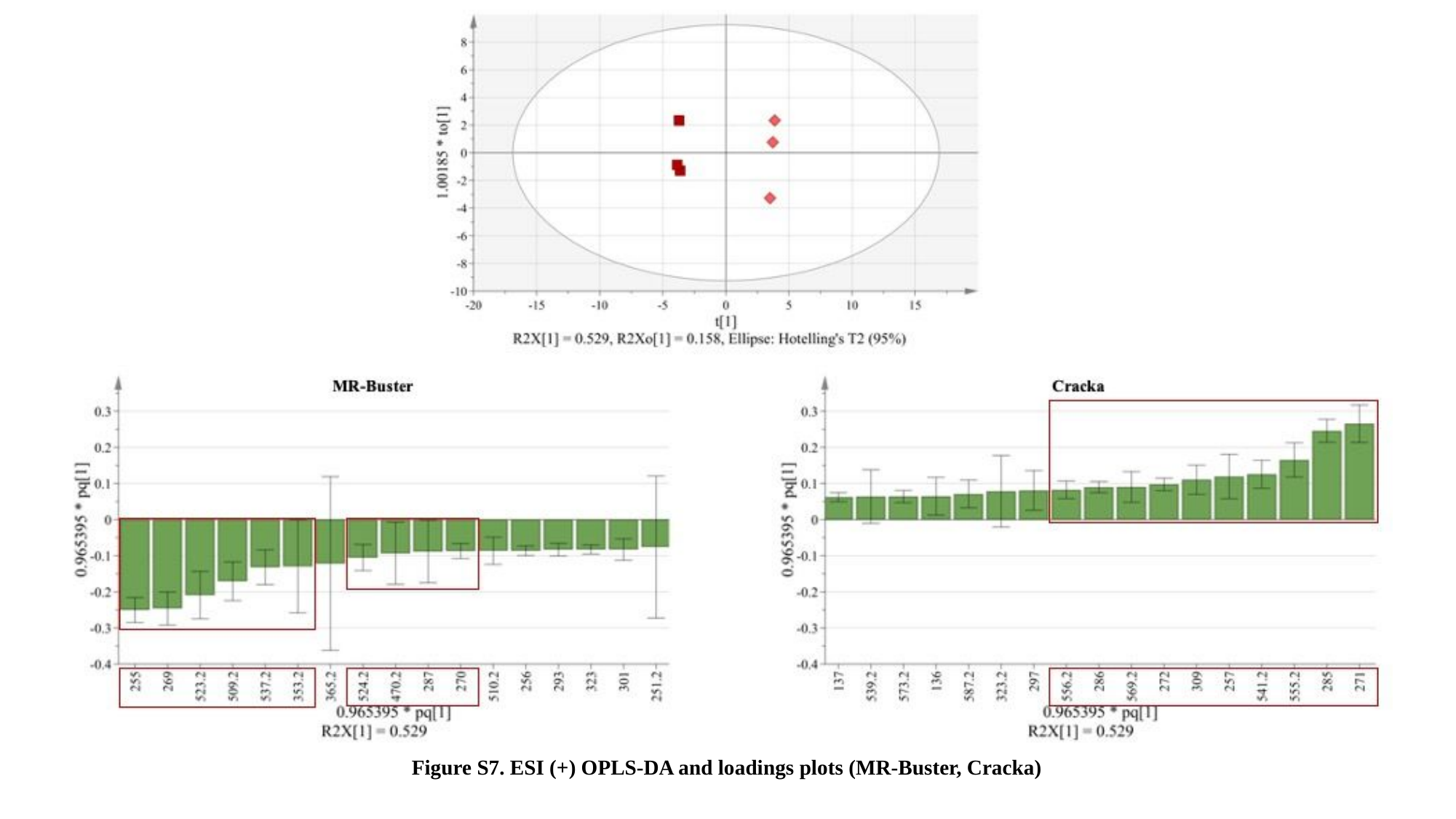

Figure S7. ESI (+) OPLS-DA and loadings plots (MR-Buster, Cracka)

## Slide 22
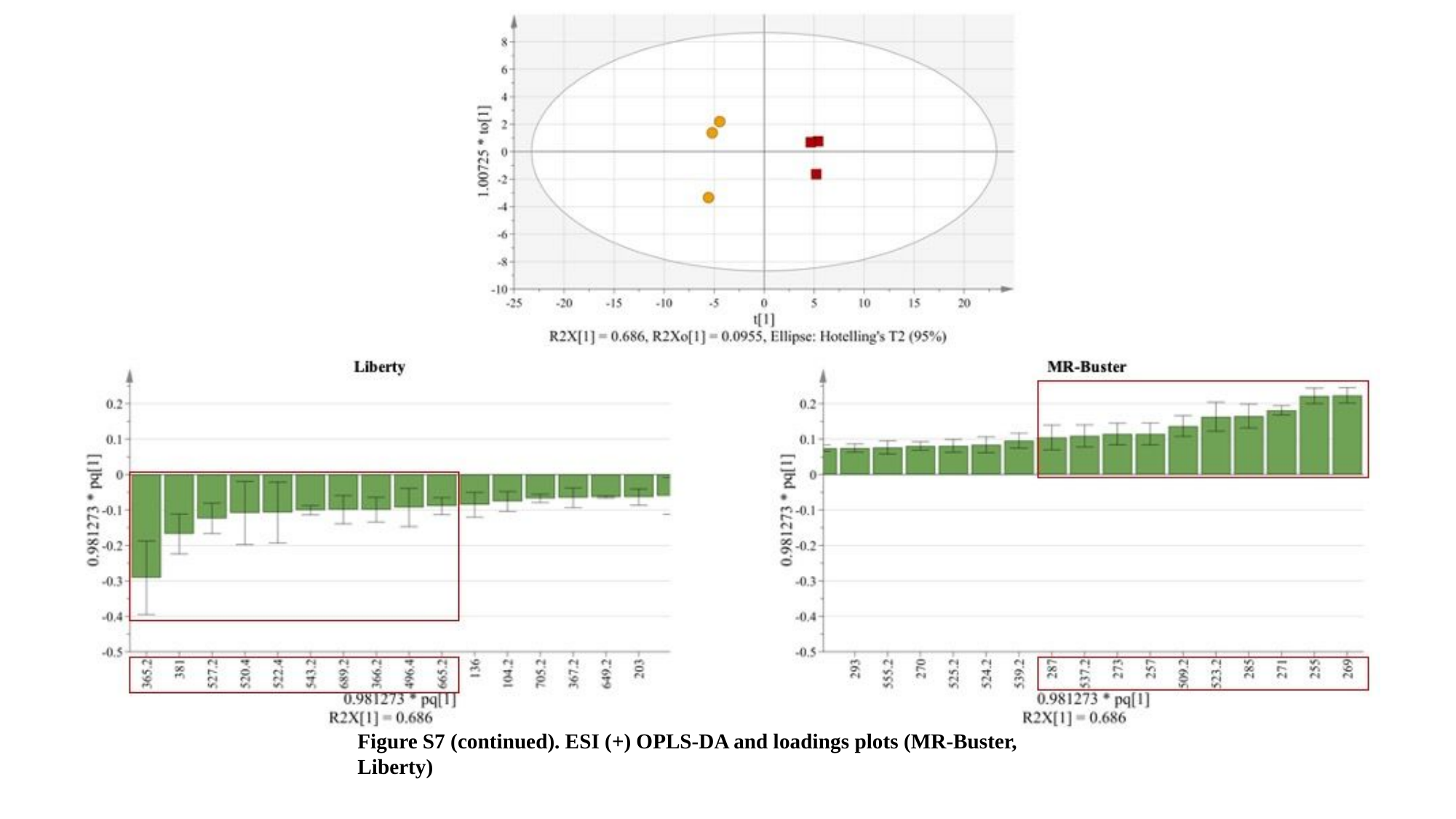

Figure S7 (continued). ESI (+) OPLS-DA and loadings plots (MR-Buster, Liberty)

## Slide 23
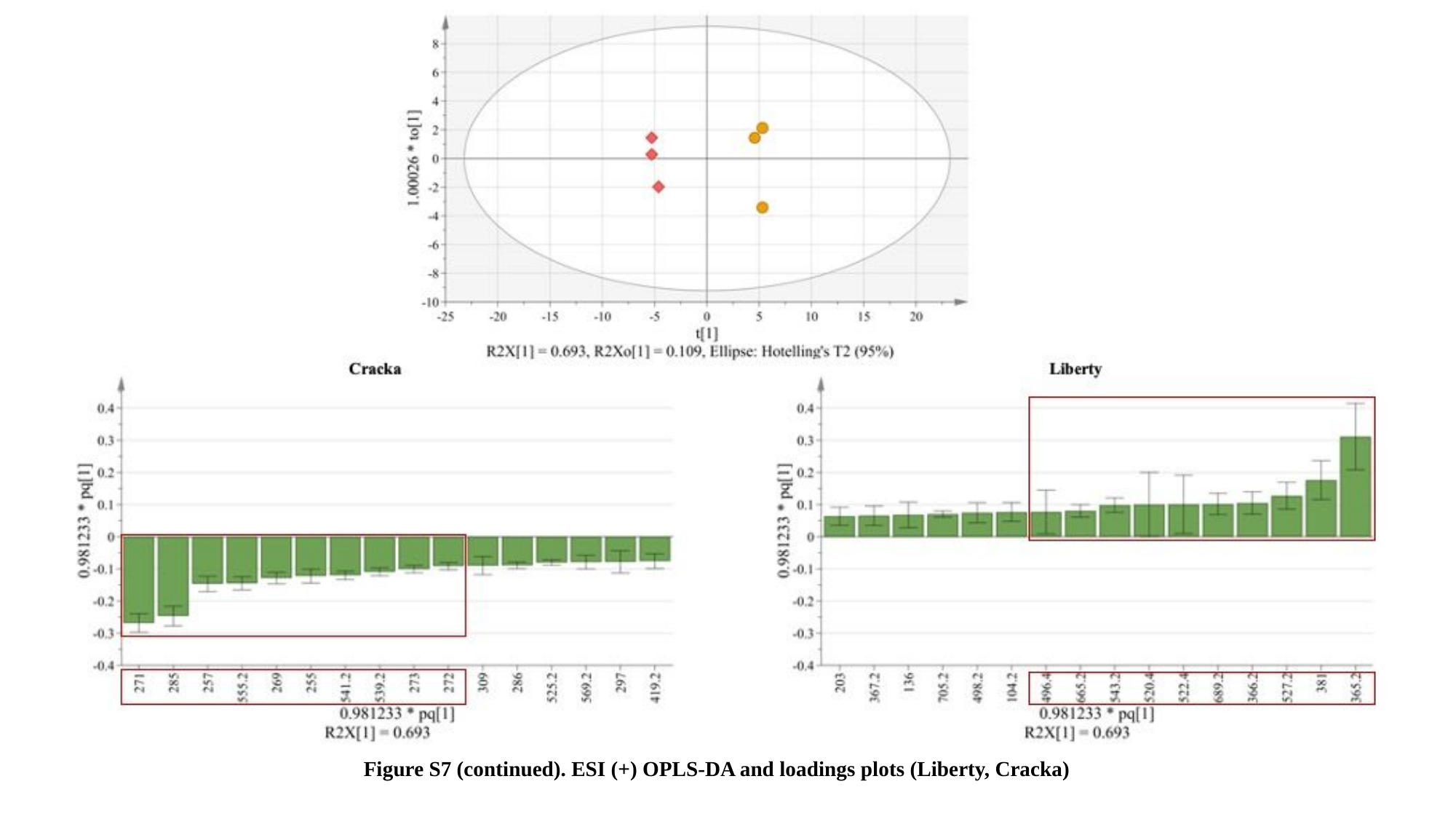

Figure S7 (continued). ESI (+) OPLS-DA and loadings plots (Liberty, Cracka)

## Slide 24
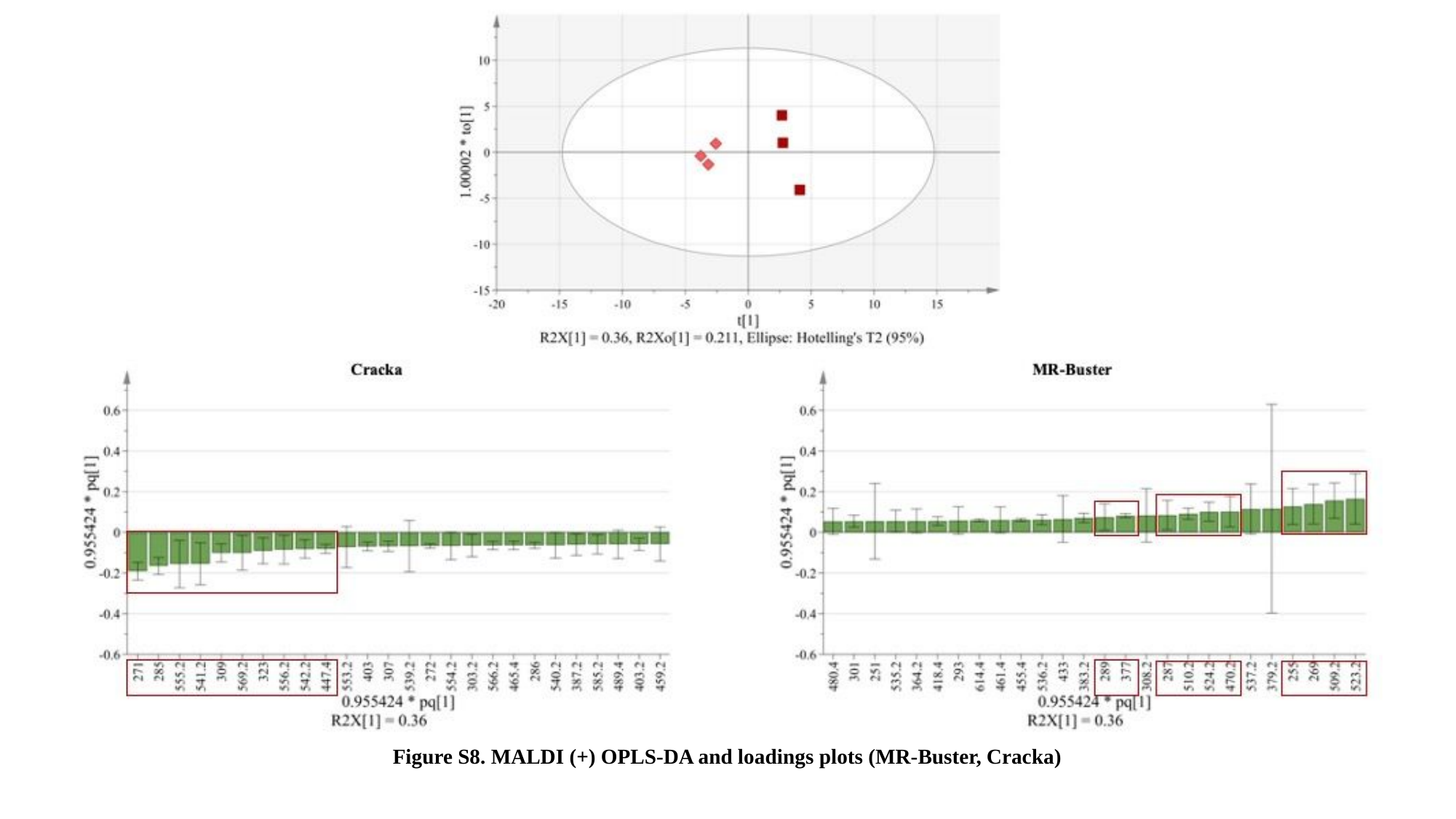

Figure S8. MALDI (+) OPLS-DA and loadings plots (MR-Buster, Cracka)

## Slide 25
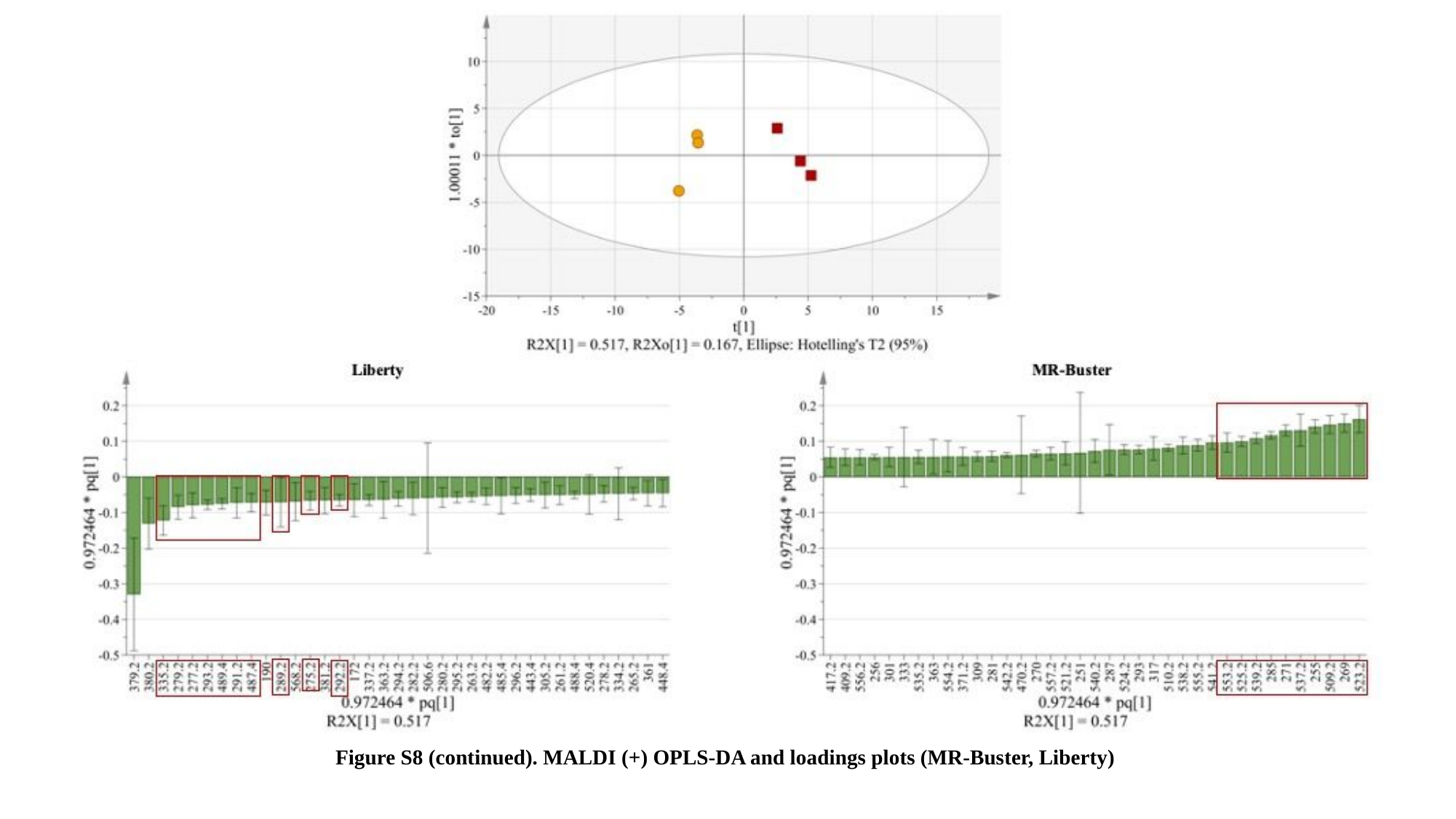

Figure S8 (continued). MALDI (+) OPLS-DA and loadings plots (MR-Buster, Liberty)

## Slide 26
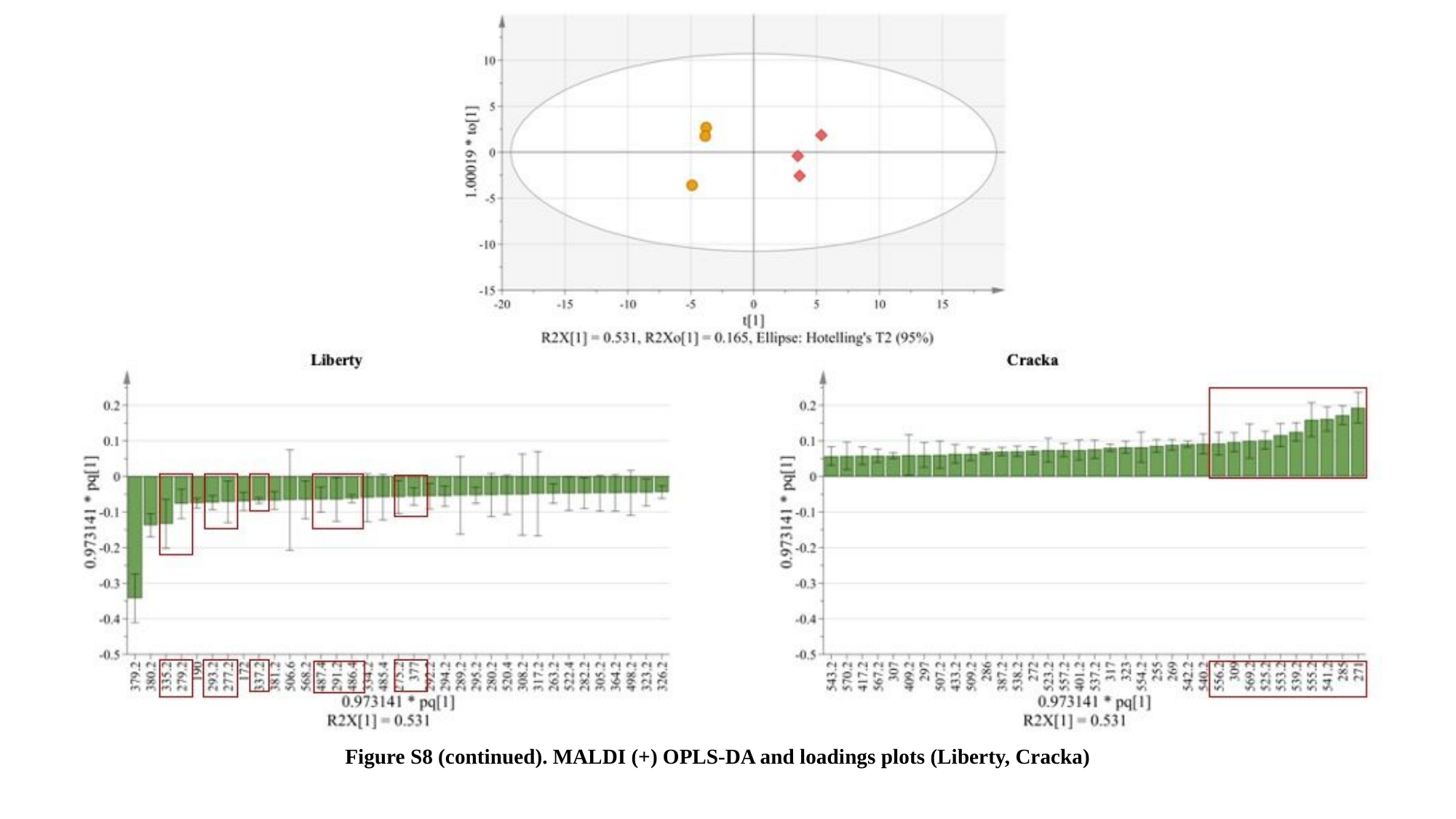

Figure S8 (continued). MALDI (+) OPLS-DA and loadings plots (Liberty, Cracka)

## Slide 27
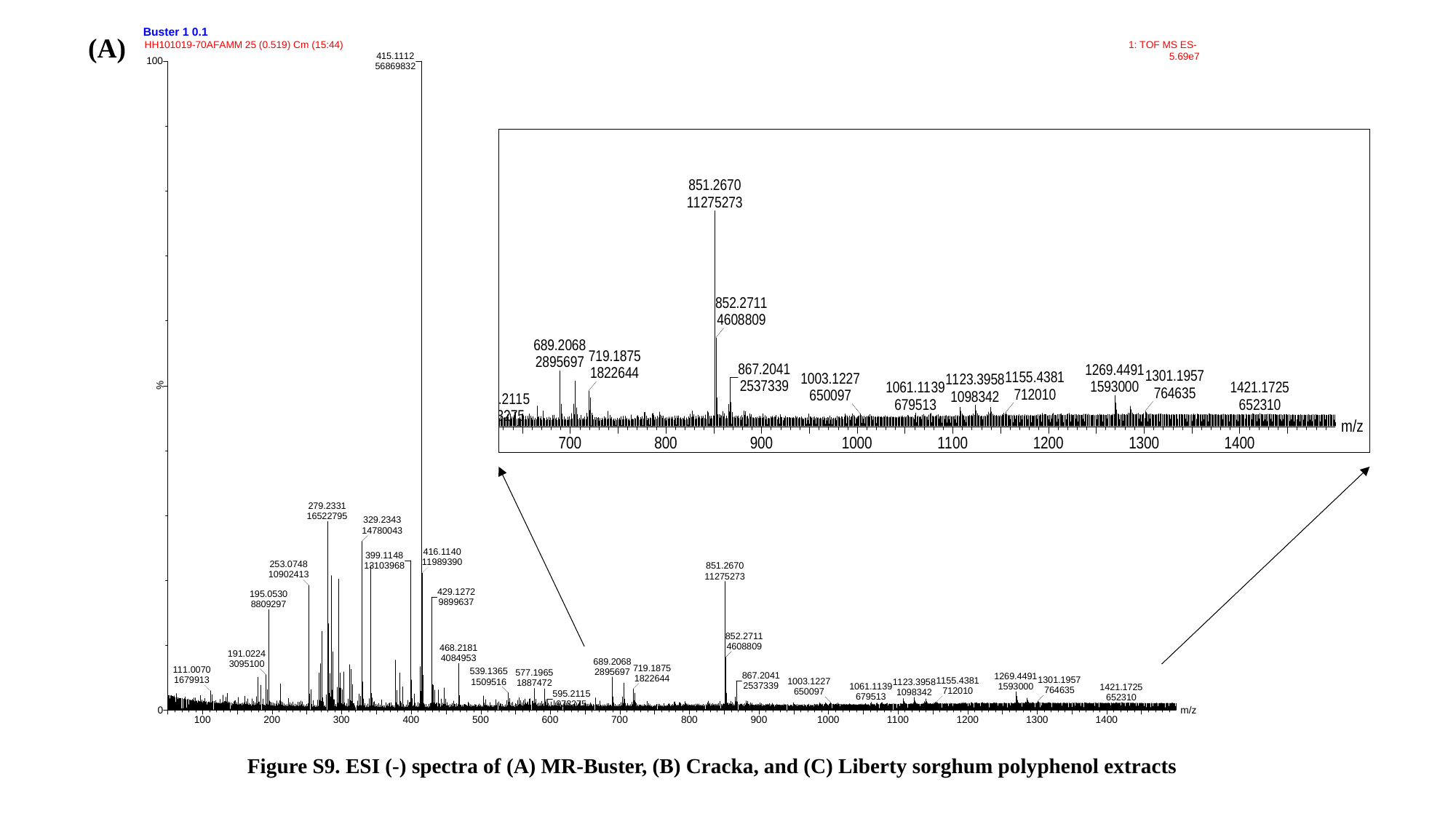

(A)
Figure S9. ESI (-) spectra of (A) MR-Buster, (B) Cracka, and (C) Liberty sorghum polyphenol extracts

## Slide 28
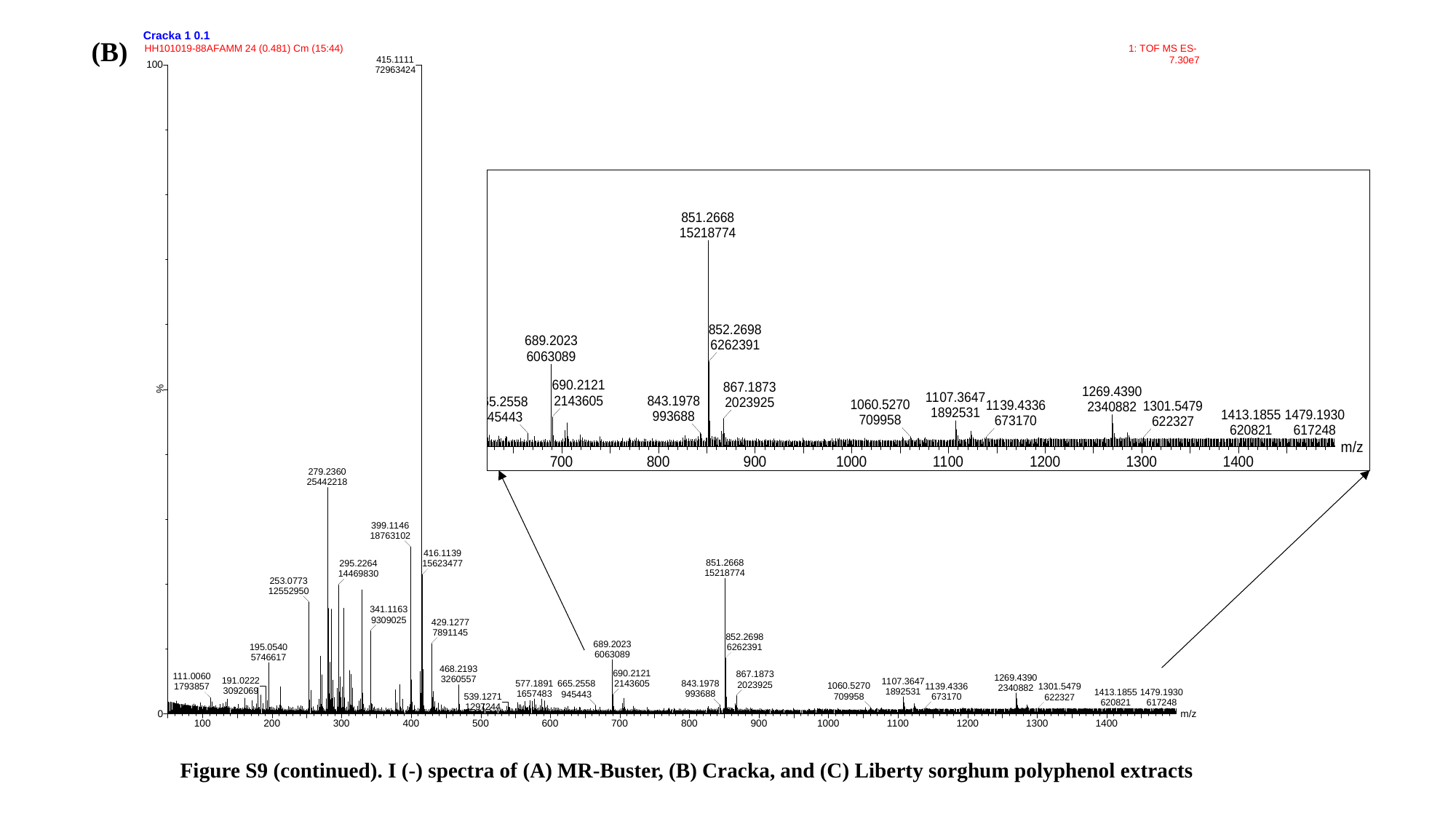

(B)
Figure S9 (continued). I (-) spectra of (A) MR-Buster, (B) Cracka, and (C) Liberty sorghum polyphenol extracts

## Slide 29
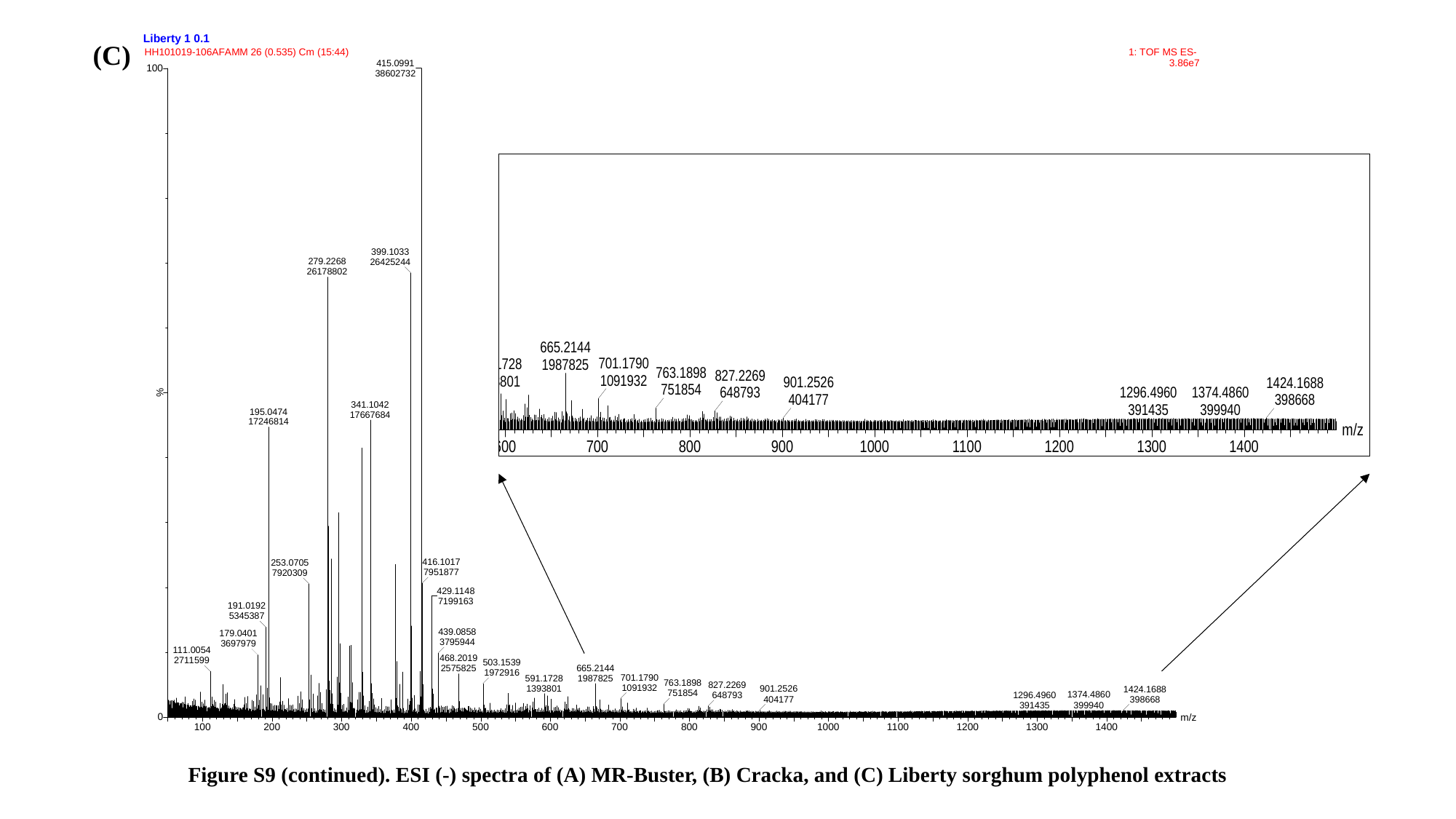

(C)
Figure S9 (continued). ESI (-) spectra of (A) MR-Buster, (B) Cracka, and (C) Liberty sorghum polyphenol extracts
